# *Physalis floridana Cell Number Regulator1* encodes a cell membrane-anchored modulator of cell cycle and negatively controls fruit size

**DOI:** 10.1093/jxb/eru415

**Published:** 2014-10-11

**Authors:** Zhichao Li, Chaoying He

**Affiliations:** ^1^State Key Laboratory of Systematic and Evolutionary Botany, Institute of Botany, Chinese Academy of Sciences, Nanxincun 20, Xiangshan, Haidian, 100093 Beijing, PR China; ^2^University of Chinese Academy of Sciences, Yuquan Road 19, 100049 Beijing, PR China

**Keywords:** Berry size, cell cycle, fruit development, natural variation, *Physalis*, seed

## Abstract

*Physalis* species show a significant variation in berry size; however, the underlying molecular basis is unknown. In this work, we showed that cell division difference in the ovaries might contribute to the ultimate berry size variation within *Physalis* species, and that mRNA abundance of *Physalis floridana Cell Number Regulator1* (*PfCNR1*), the putative orthologue of the tomato *fruit weight 2.2* (*FW2.2*), was negatively correlated with cell division in the ovaries. Moreover, heterochronic expression variation of the *PfCNR1* genes in the ovaries concomitantly correlated with berry weight variation within *Physalis* species. In transgenic *Physalis*, multiple organ sizes could be negatively controlled by altering *PfCNR1* levels, and cell division instead of cell expansion was primarily affected. PfCNR1 was shown to be anchored in the plasma membrane and to interact with PfAG2 (an AGAMOUS-like protein determining ovary identity). The expression of *PfCYCD2;1*, a putative orthologue of the mitosis-specific gene *CyclinD2;1* in the cell cycle was negatively correlated with the *PfCNR1* mRNA levels. PfAG2 was found to selectively bind to the CArG-box in the *PfCYCD2;1* promoter and to repress *PfCYCD2;1* expression, thus suggesting a PfAG2-mediated pathway for PfCNR1 to regulate cell division. The interaction of PfCNR1 with PfAG2 enhanced the repression of *PfCYCD2;1* expression. The nuclear import of PfAG2 was essential in the proposed pathway. Our data provide new insights into the developmental pathways of a cell membrane-anchored protein that modulates cell division and governs organ size determination. This study also sheds light on the link between organ identity and organ growth in plants.

## Summary

Highlight: A cell membrane-anchored protein governs ovary cell division and determines berry size mediated by a MADS-domain protein, suggesting a molecular link between ovary identity and growth in plants.

## Introduction

The genetic mechanism underlying natural variations in plant organ size between species and within species has been elusive. Fruit size is an important agronomic trait and is the prime criteria for domestication. Developmental comparisons have suggested that, among equivalent (orthologous) organs from plant species of different size, larger organs result mainly from increased cell number rather than from larger cells (Mizukami, 2001). The Solanaceae family is a source of nutrition and culinary diversity for human populations. Five of the leading economic species in this family are potato (*Solanum tuberosum*), tomato (*Solanum lycopersicum*), eggplant (*Solanum melongena*), pepper (*Capsicum* spp.), and husk tomato (*Physalis* spp.). A number of key loci controlling fruit size and a subset of genes underlying these loci have been studied in this plant family, such as *Fruit weight2.2* (*FW2.2*), *fasciated* (*FAS*), and *locule number* (*LC*) (Frary *et al.*, 2000; Cong *et al.*, 2008; Muños *et al.*, 2011).

*FW2.2* is the first quantitative trait locus (QTL) cloned in plants. The mutation in *FW2.2* is supposed to be the first step in the domestication of larger tomato fruit, and *FW2.2* alone controls up to 30% of fruit weight variation (Frary *et al.*, 2000). *FW2.2* encodes a repressor of cell division, and this function was fulfilled by negatively regulating expression of this gene, rather than via changes in protein structure (Frary *et al.*, 2000; Cong *et al.*, 2002). *FW2.2-like* genes have also been studied in several other species. In maize, the putative orthologue of tomato *FW2.2* is *cell number regulator1* (*CNR1*), which affects entire plant and multi-organ size by negatively regulating cell number (Guo *et al.*, 2010). The avocado (*Persea americana*) *fruit weight2.2-like* (*Pafw2.2-like*) gene has been proposed to play a conserved role as a negative regulator of fruit cell division (Dahan *et al.*, 2010). In soybean (*Glycine max*), *fruit weight2.2-like1* (*GmFWL1*) is a homologue of the tomato *FW2.2* gene and was found to be essential for soybean nodule organogenesis as a result of effects on plant cell division (Libault *et al.*, 2010). In rice (*Oryza sativa*), *fruit weight2.2 like3* (*Osfwl3*) controls the grain weight by negatively regulating cell division (Xu *et al.*, 2013). *Prunus avium cell number regulator* (*PavCNR*), which is a homologue of the tomato *FW2.2*, is a cell number regulator gene in *Prunus* species and associates with fruit size in sweet and sour cherry (Franceschi *et al.*, 2013). *FW2.2-like* genes play a conservative role in cell division in different species (Guo and Simmons, 2011; van der Knaap *et al.*, 2014). However, they have been found to encode cell membrane-anchored proteins (Cong and Tanksley, 2006; Libault *et al.*, 2010; Xu *et al.*, 2013), and how they regulate cell division is an intriguing question. Little is known about the molecular and biochemical role of these proteins. FW2.2-like proteins were found to facilitate iron transport (Song *et al.*, 2004; Nakagawa *et al.*, 2007), but the role of iron change in regulating cell division is not established yet. Further yeast two-hybrid screens using *FW2.2* as baits revealed that the encoded protein interacts with the regulatory subunit of casein kinase II (CKII) (Cong and Tanksley, 2006), a protein with broad activity that includes the control of cell division (Pepperkok *et al.*, 1994; Espunya *et al.*, 1999; Moreno-Romero *et al.*, 2008), providing a step forward to understand the role of FW2.2 in cell division. However, details of the developmental pathway of FW2.2-like proteins that regulate cell division are largely unknown.

**Fig. 1.**
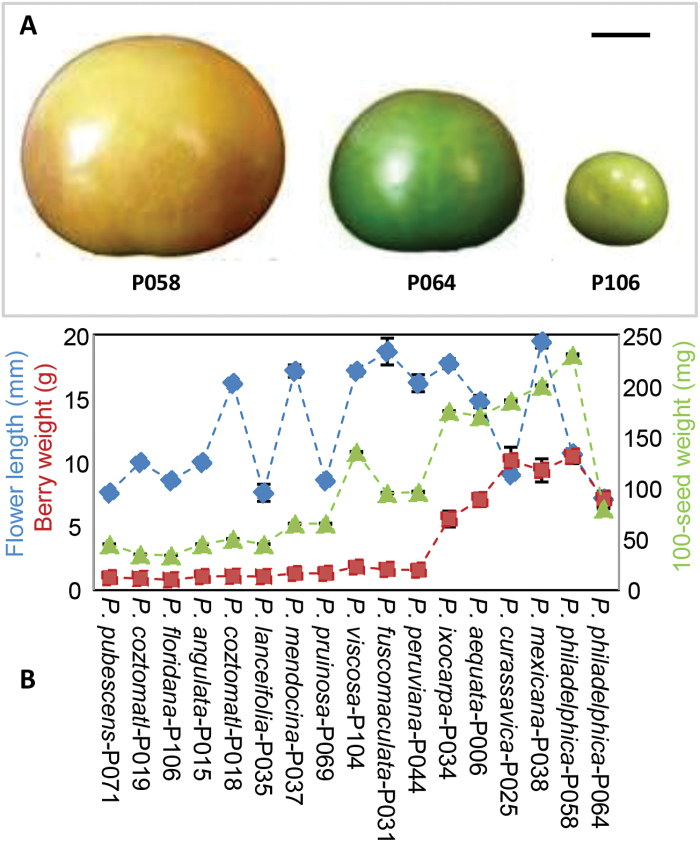
Organ size variation in *Physalis* species. (A) Mature berry in *Physalis*. Bar, 1cm. From left to right: *P. philadelphica* (P058), *P. philadelphica* (P064), and *P. floridana* (P106). The calyces were removed. (B) Size variation of flowers (blue), berries (red) and seeds (green) in 16 *Physalis* species. One accession for 15 species and two accessions of *P. philadelphica* (P058 and P064) were included and their information is shown in Supplementary Table S1 at *JXB* online. The dashed linkage of each accession is for clarity only. The mean and standard deviation are presented.

The genus *Physalis* has more than 70 species and has become a new model to study the evolution and developmental control of morphological novelty (He *et al.*, 2004; He and Saedler, 2005; Wang *et al.*, 2012; Zhao *et al.*, 2013) because a *Physalis* fruit features a distinct fruiting clayx called ‘inflated calyx syndrome’ (ICS) or the ‘Chinese lantern’. However, study of the developmental and molecular control of *Physalis* berry size has long been neglected. *Physalis* species have a rich diversity in berry size (Fig. 1), and a few *Physalis* species have been cultivated for the production of the berries, for example *Physalis philadelphica* (Montes Hernández and Aguirre Rivera, 1994). Most of the species are diploid (2*n*=24), except for *Physalis peruviana*, which is tetraploid (Sinha, 1951; He and Saedler, 2005; Wang *et al.*, 2012). The berry size seems to be uncoupled with the polyploidy level within *Physalis* species, but expression variation of several genes during flower and berry development might contribute to the berry size variation (Wang *et al.*, 2012). In the present study, we showed that *Physalis floridana Cell Number Regulator1* (*PfCNR1*), a putative orthologue of the tomato *FW2.2*, encodes a cell membrane-anchored protein and functions as a negative regulator of cell division. We further showed that *PfCNR1* is involved in the cell division cycle through molecular interactions of PfCNR1 with *P. floridana* AG2 (PfAG2, an AGAMOUS-like MADS-domain regulatory protein) and of PfAG2 with *P. floridana CyclinD2;1* (*PfCYCD2;1*, a putative *CyclinD2;1* gene that encodes a key component at the G_1_/S phase in the cell cycle), thus directing cell division and contributing to natural variation of berry size within the *Physalis* species. Our work may also provide a crucial mechanistic link between organ patterning and growth.

## Materials and methods

### Plant materials

The *Physalis* resources (Supplementary Table S1) were grown in a greenhouse at the Institute of Botany, Beijing, China. The stage of the mature flower was set as d 0. Flower buds at 9 (B1), 6 (B2), and 3 (B3) d before anthesis and mature flowers (F), as well as 5, 10, 15, 20, 25, 30, and 50 day post-anthesis (DPA) fruits and leaves, and seeds from 15 and 30 DPA fruits of *P. floridana* (P106), *P. philadelphica* (P064) and *P. philadelphica* (P058) were collected. The B2 flower buds of all species/accessions were harvested for quantitative transcript analysis.

### Trait quantification of *Physalis* species

Three plants for each *Physalis* species/accession were transplanted into the experimental field in the summers of 2009, 2010, and 2012. The size of the mature flower was represented by the length between the receptacle and the tip of mature flowers. Mature berry size was defined by the weight, and the 100-seed weight was measured. For each plant/accession, the size of 15–60 mature flowers and 10–100 mature berries was quantified. Some accessions/species did not grow well in all 3 years, and the traits of these accessions were quantified based on 1- or 2-year observations. The seed weight was measured using three independent batches of 100 seeds for each species.

### Cell number and size measurement

Ovaries from flower buds (B1, B2, and B3), mature flowers (F), and 5 DPA and 50 DPA berries (berries were cut longitudinally into eight equal parts and the seeds were stripped out) and the seeds of 50 DPA berries were fixed with 3.7% formaldehyde, 5% glacial acetic acid and 50% ethanol (FAA) for 48h at room temperature. The median transverse section area and cell size were determined by AxioVision Rel. 4.7 and cell number was counted manually as described previous (Frary *et al.*, 2000; Cong *et al.*, 2002). Five median transverse sections for each stage sample were analysed. The cell size of 150 cells for each tissue on each section was measured.

### Quantitative transcript analyses

Total RNAs were isolated with a Plant Total RNA Extraction kit (Bioteke) and treated with RNase-free DNase I (Promega). First-strand cDNA was synthesized by reverse transcription with oligo(dT)_17_ using SuperScript^®^ III Reverse Transcriptase (Invitrogen). The transcript abundance of genes was quantified using quantitative reverse transcription (qRT)-PCR via the SYBR^®^ *Premix Ex Taq*™ kit (Takara). *PfACTIN* was used as an endogenous control. All PCR reactions were run on a Mx3000P^TM^ Real Time PCR thermal cycler using the gene-specific primers (Supplementary Table S2 at *JXB* online).

### RNA *in situ* hybridization

Floral buds B1, B2, and B3 as well as the ovaries from P106, P058, and P064 were fixed with FAA, dehydrated in a graded ethanol series, and embedded in Paraplast (Sigma). To generate probes for hybridization, a 269bp *PfCNR1* cDNA fragment was designed as a template. Probes were synthesized using T7 RNA polymerase driven by a T7 promoter and were labelled with digoxigenin using a DIG-RNA labelling kit (Roche). Hybridization was performed as described previously (Carr and Irish, 1997), except that slides were washed in 2× SSC, 1× SSC, and then 0.5× SSC at 50 °C for 30min, respectively.

### Yeast two-hybrid assays

Total RNA mixture from stems, leaves, flower buds (different developmental stages), mature flowers, and young fruits (with calyx) of *Physalis* species was used to construct a cDNA library (He *et al.*, 2007). The baits PfCNR1, PfCNR1_1–42_, and PfCNR1_103–175_ were transformed into *Saccharomyces cerevisiae* Y187 and used to screen the pGADT7-cDNA library. The yeast library system and procedure were similar to a previous description (Cong and Tanksley, 2006). To confirm the protein–protein interaction, the open reading frames (ORFs) of PfCNR1 and its putative interacting proteins were respectively inserted into pGBKT7 and pGADT7. The selective medium, detailed information of the *Physalis* library, screen procedures, yeast co-transformation, and the non-lethal galactosidase activity assays as described previously (He *et al.*, 2007).

### Yeast one-hybrid assays

The involved DNA fragments were respectively cloned into the pAbAi vector. pGADT7-PfAG2 and pGADT7-PfSEP1 were respectively transformed into the Y1HGold yeast strain that contained each derived pAbAi construct. Protein and DNA interaction was revealed by yeast cell survival on SD/–Leu medium supplemented with 200ng ml^–1^ of aureobasidin A (AbA). The yeast one-hybrid assay was performed following the manual of the Matchmaker Gold Yeast One-Hybrid System (Clontech).

### Protein transient expression assays in plant cells

For subcellular localization, the ORFs of the involved genes were cloned into the expression vector Super1300 (Chen *et al.*, 2009) and fused with the green fluorescence protein (GFP) gene. These derived constructs were transformed into *Agrobacterium tumefaciens* LBA4404, and the leaf epidermal cells of *Nicotiana benthamiana* were infected via *Agrobacterium* infiltration. For plasmolysis, 0.3g ml^–1^ of sucrose was injected into tobacco leaves expressing PfCNR1–GFP prior to observation. The detection of GFP indicated the subcellular localization of the protein we were interested in. For bimolecular fluorescence complementation (BiFC) assays, the ORFs of the involved genes were cloned into the pair of vectors pSPYNE-35S and pSPYCE-35S (Walter *et al.*, 2004), which contained the N-terminal half (YFPn) and the C-terminal half (YFPc), respectively, of yellow florescence protein (YFP). These derived constructs were transformed into *A. tumefaciens* GV3101, and the construct recombination was injected into leaf epidermal cells of *N. benthamiana* via *Agrobacterium* infiltration. The detection of YFP signal indicated that the two proteins interacted in plant cells. The GFP or YFP signal was detected 48h after injection under a confocal laser-scanning microscope (Olympus FV1000MPE). Each portion of PfCNR1, combinations of these portions, and the deletion mutations of *PfCNR1* and *PfAG2* were introduced using a PCR approach.

### Transient luciferase (LUC) activity assays in *P. floridana* protoplasts

To produce the LUC reporter gene constructs, the indicated fragments were ligated into the YY96 vector (Yamamoto and Deng, 1998). The ORF of *PfCNR1*, *PfAG2*, *PfAG2m* (the encoded nuclear localization signal was deleted), *PfSEP1*, and *PfCKIIβ1* was ligated into the pGFP221 vector (Han *et al.*, 2005). Protoplasts of *P. floridana* (wild type, *35S:PfCNR1* OE9, and *35S:PfCNR1*-RNAi R9) were prepared from young leaves, and transient expression assays were performed as described previously (Yamamoto and Deng, 1998). Each obtained reporter plasmid as well as the *35S:GUS*- and/or pGFP221-derived constructs indicated were co-transformed into protoplasts, which were pelleted and resuspended in cell culture lysis reagent (Promega). The β-glucuronidase (GUS) fluorescence was measured using a Modulus luminometer/fluorometer with a UV fluorescence optical kit, and the LUC activity was detected with a luminescence kit (Promega). The relative LUC expression was expressed as the LUC/GUS ratio.

### Expression vector construction and *P. floridana* transformation

A double-stranded RNA interference (RNAi) construct was assembled by introducing a 229bp fragment of *PfFCNR1* cDNA into the pFGC1008 vector (He and Saedler, 2005). For overexpression, the ORF of *PfCNR1* cDNA was cloned into the vector pRT100, and the *35S:PfCNR1* cut from the derived pRT100 was introduced into the pBAR-A vector (He and Saedler, 2005). All transgenic analyses were performed in *P. floridana* P106. Transformation was performed as described previously (He and Saedler, 2005). One of the plants derived from independent explants was kept. Nine independent transgenic lines for both RNAi and overexpression were created and analysed. The genotypes of the T_2_ transgenic *Physalis* lines were verified by qRT-PCR and semi-quantitative RT-PCR. In the virus-induced gene silencing (VIGS) analysis, the fragments of *PfCNR1*, *PfCNR1L1*, and *PfCNR1L2* were introduced into the tobacco rattle virus system, which was then applied to the leaves of 2-week-old *Physalis* seedlings (Zhang *et al.*, 2014). Half-flowers (halves of sepal, petal, and stamen) were collected for qRT-PCR, and the carpels were kept intact and labelled for berry weight determination. Half-flowers of the wild type were included as controls.

### Phenotyping the transgenic *P. floridana*

Phenotypic variation of the T_2_ transgenic *Physalis* plants was analysed in comparison with the wild type. Five seeds of each T_1_ transgenic line were germinated, and at least three transgenic siblings of each transgenic line were included for phenotypic proofs. The segregating wild-type-like siblings of some transgenic lines were also analysed. The area of the sixth mature leaf (above the cotyledons) was measured. Flower length was recorded as the length between the receptacle and the petal tip. ICS area and weight of ovary, mature berry, and 100 seeds were measured. Epidermal cells of leaves, sepals, and ICS were imaged using scanning electron microscopy. Ovaries from mature flowers and mature berries of transgenic plants were made into paraffin sections for evaluating cell division and cell expansion in comparison with those of the wild type.

### Statistical analyses

The correlation coefficient (*r*), a *t*-test, and the associations of three quantitative traits (berry weight, 100-seed weight, and flower length) with the variation in amino acid sites and with the variation in gene expression were analysed using SPSS 15.0.

### Sequencing analyses

Using tomato *FW2.2* to search the Tomato Genome Sequencing Project (http://mips.helmholtz-muenchen.de/plant/tomato/index.jsp) and the NCBI database (http://www.ncbi.nlm.nih.gov/), we identiﬁed another two sequences that were closely related to *FW2.2* homologous genes (*FW2.2L1* and *FW2.2L2*). Their orthologous cDNAs (*PfCNR1*, *PfCNR1L1*, and *PfCNR1L2*) in P106 were isolated using a 5’ and 3’ Rapid Amplification of cDNA Ends kit (Roche). Total DNA from the leaves of P106, P058, and P064 was extracted using a Plant Genomic DNA kit (Tiangen). Genomic sequences were isolated using rapid amplification of genomic DNA ends according to a Universal Genome Walker kit (Clontech). All primers used in the present work are shown in Supplementary Table S2. The phylogenetic trees were reconstructed under default parameters using the MEGA5 tool (Tamura *et al.*, 2011). The transmembrane domain of PfCNR1 was predicted using the TopPred program online (http://www.sbc.su.se/~erikw/toppred2/). The nuclear localization signal (NLS) of PfAG2 was predicted by the cNLS Mapper (http://nls-mapper.iab.keio.ac.jp/). Sequence data from this study have been deposited in GenBank under accession numbers KJ155732–KJ155748.

## Results

### Berry size variation within the *Physalis* species

We collected 16 *Physalis* species (Supplementary Table S1). Considerable variations in the berry size among these *Physalis* species were observed, as seen in P058 (*P. philadelphica*), P064 (*P. philadelphica*), and P106 (*P. floridana*), which produced berries with different sizes (Fig. 1A). The berry weight (designated berry size) varied from 0.86±0.30g in *P. floridana* P106 to 11.0±0.60g in *P. philadelphica* P058 within the 16 *Physalis* species (Fig. 1B). The flower length and seed weight also varied significantly among these *Physalis* species (Fig. 1B). The flower length was recorded by the length between the receptacle and the tip of mature flowers. We observed that flower length varied from 7.5±0.2mm in *P. philadelphica* P064 to 20.4±0.5mm in *P. Mexicana* P038. The 100-seed weight was also measured for each species and ranged from 34±0.1mg in *P. floridana* P106 to 230±1.0mg in *P. philadelphica* P058. Unlike the observations in *P. philadelphica* (Wang *et al.*, 2012), a correlation between flower length and berry weight was not observed among these *Physalis* species (*r*=0.05, *P*=0.84), nor between flower length and 100-seed weight (*r*=0.38, *P*=0.13). Interestingly, a positive correlation between berry weight and 100-seed weight was seen (*r*=0.88, *P*<0.01). Thus, among these traits, berry size is the prime breeding target for *Physalis* crops.

### Dynamics of cell activity during *Physalis* berry development

**Fig. 2.**
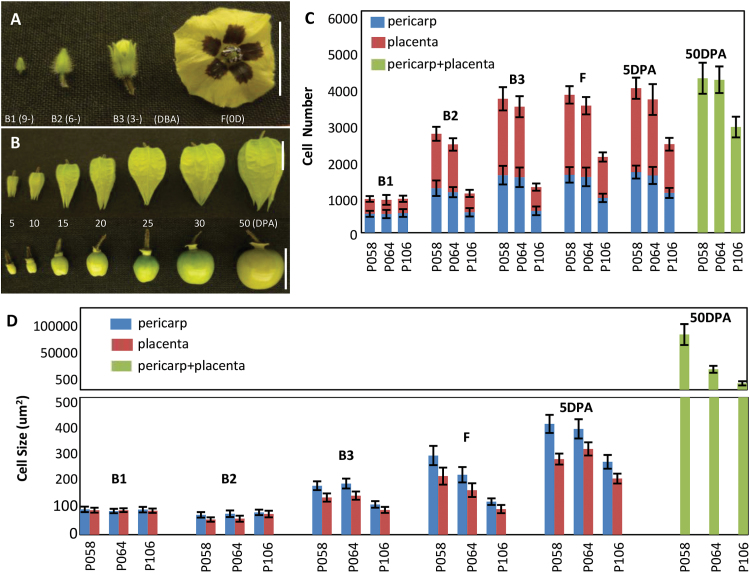
Berry development in *Physalis* species. (A) Flower development. The developmental stage of the mature flower (F) was set as d 0, and the floral buds at 9 (B1), 6 (B2), and 3 (B3) d before anthesis (DBA) are presented. (B) Fruit development. The developing berries of 5, 10, 15, 20, 25, 30, and 50 DPA are presented. The top images are intact fruits, and the bottom images represent the berries in which the inflated calyx was removed. The developmental stages in *P. philadelphica* were similarly defined for harvesting of tissues. Bars, 1cm. (C) Cell number variation. (D) Cell size variation. Quantification was performed according to median transverse section of the developing carpel and fruit from the floral buds (B1, B2, and B3), mature flower (F) and the developing berries of 5 and 50 DPA (see Materials and Methods for details). Five median transverse sections for each stage sample were analysed. The cell size of 150 cells for each tissue on each section was measured. The cell number for each section was counted. The mean and standard deviation are presented. The details are presented in Tables S3 and S4.

The berry expands from the ovary in response to fertilization. Cell division and cell expansion are believed to be responsible for the growth of the berry. To reveal any potential differences in these cellular levels among *Physalis* species, we decided to evaluate the cell division and cell expansion in ovary and developing berries. The mature flower (F) stage was set as d 0, and the developmental stages of a *Physalis* ovary and berry included 9 (B1), 6 (B2), and 3 (B3) d before anthesis, and 5, 10, 15, 20, 25, 30 and 50 DPA, which were exemplified by *P. floridana* (Fig. 2A, B). Median transverse sections of developing ovaries and berries were subjected to further evaluations. We found that the ovary size and the increase in the flesh (pericarp and placenta) size in developing berries might largely determine the mature berry size (Supplementary Fig. S1A, B, at *JXB* online). This was corroborated by the fact that cell division in the ovaries from floral buds (B1, B2, and B3) to mature flowers (F) was more active than that in the flesh of 5 and 50 DPA berries; however, cell division in the ovules and the developing seeds increased rapidly as the seeds developed (Fig. 2C; Supplementary Table S3 at *JXB* online). The different cell expansion from the B2 ovary stage to mature berries in the *Physalis* species might explain the variation in mature berry size, whereas different cell expansion in the fertilized ovules might explain the seed weight (Fig. 2D; Supplementary Table S4 at *JXB* online).

Comparison analyses among *Physalis* species (Fig. 2C, D, and Supplementary Tables S3 and S4) suggested that the cell division was undistinguished in different accessions (P058 and P064) of *P. philadelphica*, and thus berry size variation in the species was due to the difference in cell expansion originating in the B2 ovaries. Nonetheless, cell division activity in the ovaries was significantly different prior to anthesis in *P. philadelphica* (P058 and P064) and *P. floridana* (P106), partly contributing to berry size variation.

### Characterization of the *PfCNR1* gene family in *Physalis* species

**Fig. 3.**
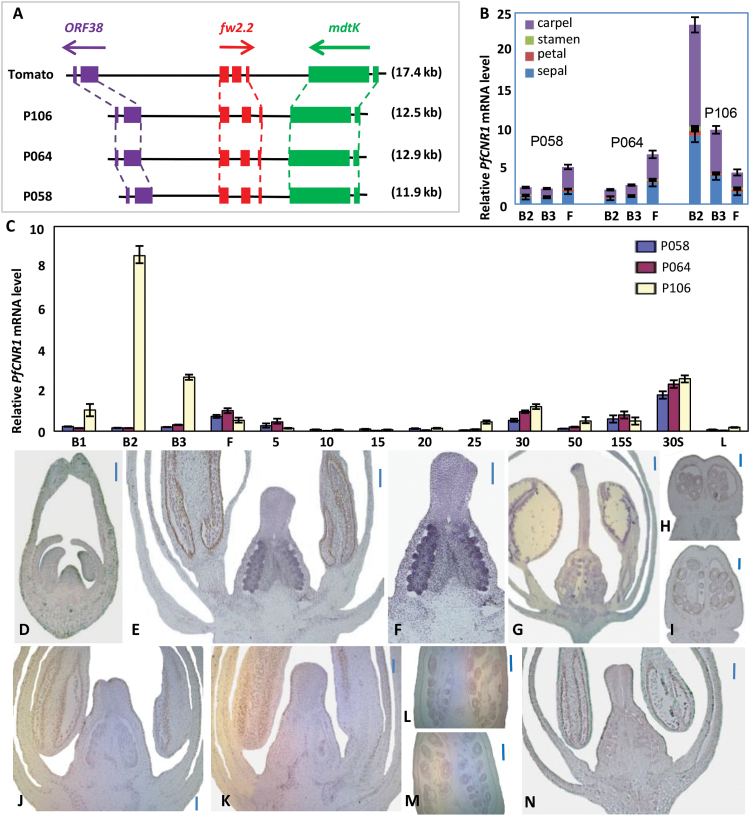
Characterizations of the *PfCNR1* gene family. (A) Local synteny of *FW2.2* and *PfCNR1* between tomato and *Physalis* species. *PfCNR1* genes were isolated from *P. philadelphica* (P058), *P. philadelphica* (P064), and *P. floridana* (P106). The upstream *ORF38* of *FW2.2* (*PfCNR1*) encodes an unknown protein, while the downstream gene is *mdtK*. The full length of each assembled genomic fragment is given in parenthesis. (B) *PfCNR1* expression in four floral whorls at anthesis. *P. philadelphica* (P058), *P. philadelphica* (P064), and *P. floridana* (P106) were tested. (C) *PfCNR1* expression in *Physalis* species. Developmental stages were defined as in Fig. 2A and B. 15S, seeds from 15 DPA; 30S, seed from 30 DPA; L, leaf. *PfACTIN* was used as an internal control. The experiments were performed using three independent biological samples. The mean and standard deviation are presented. (D–i) *In situ* hybridization of *PfCNR1* in *P. floridana* P106: B1 flower bud (D); B2 flower bud (E); carpel of the B2 flower bud (F); B3 flower bud (G); carpel of the mature flower (H); 5 DPA fruit (I). (J–M) *In situ* hybridization of *PfCNR1* in *P. philadelphica* P058 and P064: B2 flower bud of P058 (J); B2 flower bud of P064 (K); carpel of the mature flower of P058 (L); carpel of the mature flower of P064 (M). A *PfCNR1* anti-mRNA probe was used in (D)–(M). (N) *In situ* hybridization of *PfCNR1* in a B2 flower bud of *P. floridana* P106 using a *PfCNR1* sense probe as a negative control. Bars, 100 μm. Developmental stages were identical as defined in Fig. 2A and B.

*FW2.2* controls tomato size mainly by regulating carpel cell division (Frary *et al.*, 2000). We therefore focused on characterizing the homologous genes in *Physalis* species. Three cDNAs were isolated from *P. floridana* and designated as the *PfCNR1* family. *PfCNR1* encoded a protein that shared the highest identity (80%) with FW2.2, while the other two, which were designated as *PfCNR1*-like 1 (*PfCNR1*L1) and *PfCNR1*L2, had 52 and 50% identity, respectively, with *PfCNR1*. The two additional *FW2.2* homologous genes, *FW2.2L1* and *FW2.2L2*, were also found in the tomato genome. The orthology between *PfCNR1* and *FW2.2* was supported by phylogenetic analyses of the gene family (Supplementary Fig. S2 at *JXB* online) and local genomic microsynteny analyses (Fig. 3A). We assembled the genomic fragments harbouring the putative genes *ORF38*, *PfCNR1*, and *mdtK* through genomic walking. These were conserved among P106, P064, P058, and tomato. Considering the role of *FW2.2* in tomato (Frary *et al.*, 2000; Cong *et al.*, 2002; Liu *et al.*, 2003), we focused our investigation on the role of *PfCNR1* in *Physalis* species. The two close parologues, *PfCNR1L1* and *PfCNR1L2*, were used as controls in some cases.

### Expression of *PfCNR1* genes during flower/fruit development

We next investigated the mRNA accumulation pattern of *PfCNR1* during flower and fruit development, and found that *PfCNR1* was differentially expressed in the four whorls of *Physalis* flowers, and that it was relatively highly expressed in calyx and carpel before anthesis (Fig. 3B). In the three *Physalis* accessions, *PfCNR1* had two peaks during flower and berry development. The first peak occurred before fertilization, while the second peak appeared in the 30 DPA berries (Fig. 3C). Unlike the second expression peak, which occurred simultaneously in different species, the first *PfCNR1* expression peak occurred in the B2 stage in *P. floridana* (P106) with smaller berries. However, *PfCNR1* heterochronically peaked in the mature flowers of *P. philadelphica* (P058 and P064) that produced bigger berries (Fig. 3C). The appearance of the *PfCNR1* expression peak before anthesis occurred concomitantly with the repression of cell division activity (Fig. 3C; Supplementary Table S3). Moreover, *PfCNR1* expression levels at the B2 stage showed a negative correlation to cell division in the ovaries, while the expression of *PfCNR1L1* and *PfCNR1L2* did not correlate with the cell division activity (Supplementary Fig. S3 and Supplementary Table S5 at *JXB* online). The second *PfCNR1* expression peak was expressed mainly in the seeds (Fig. 3C). Thus, the *PfCNR1* heterochronic expression and mRNA accumulation prior to anthesis were associated with berry development.

We then conducted *in situ* hybridization to detect the cellular distribution of *PfCNR1* mRNA in the carpel. Consistent with the findings of qRT-PCR analyses (Fig. 3C), the reproducible *PfCNR1* expression signals were only detected in B2 flower buds in P106 (Fig. 3D–I). The strongest *PfCNR1* expression signals were visible in the ovules and the regions proximal to ovules in the placenta (Fig. 3E, F). The *in situ PfCNR1* expression signals in P058 and P064 were not detectable in the floral organs of similar developmental stages as *P. floridana* P106 (Fig. 3J–M) due to the extremely weak expression of this gene in *P. philadelphica* (P058 and P064) as shown in qRT-PCR analyses (Fig. 3C). The *PfCNR1* sense probe did not produce any signal in the B2 floral buds of P106 (Fig. 3N).

Therefore, differential expression of *PfCNR1* seems to be important for organ size variation. To get further clues for this, we investigated the expression of the *PfCNR1* alleles in our collected *Physalis* species using a qRT-PCR approach. We found that, among the 16 *Physalis* species, *PfCNR1* transcript levels in B2 flower buds were negatively correlated with both mature berry weight (*r*=–0.67, *P*<0.01) and 100-seed weight (*r*=–0.78, *P*<0.01) (Supplementary Table S6 at *JXB* online). We also sequenced the *PfCNR1* cDNA from the 16 *Physalis* species, and found that the encoded PfCNR1 proteins were highly conserved with nine polymorphic sites, but no significant association (*P*>0.05) was observed between sequence variations and the three quantitative traits (berry weight, 100-seed weight, and flower length) (Supplementary Table S6). All these findings suggested that altering expression of *PfCNR1* is probably involved in the variation of the fruit size. To understand better the developmental role of this gene, molecular pathways associated with *PfCNR1* were dissected in *P. floridana*, which is a well-established model of the genus *Physalis* (Zhang *et al.*, 2014).

### *PfCNR1* is involved in the control of *Physalis* berry size

VIGS analyses were first performed in *P. floridana* to quickly obtain insight into the function of these genes. Half-flowers, which included halves of calyx, corolla, and stamens, were used for studying gene expression, while the carpels were kept intact and labelled for berry size determination. Altogether, 62, 56, and 60 labelled berries were finally harvested from the VIGS plants of *PfCNR1*, *PfCNR1L1*, and *PfCNR1L2*, respectively. The berry size of the *PfCNR1*-VIGS population ranged from 0.71 to 1.31g, and the *PfCNR1* mRNA showed a significant negative correlation with the berry size (*r*=–0.52, *P*<0.001) (Supplementary Fig. S4A at *JXB* online). The berry size of the *PfCNR1L1*-VIGS and *PfCNR1L2*-VIGS populations varied from 0.74 to 1.20g and from 0.72 to 1.22g, respectively. Downregulation of either *PfCNR1L1* (*r*=–0.07, *P*>0.05) or *PfCNR1L2* (*r*=–0.09, *P*>0.05) had no effect on berry size (Supplementary Fig. S4B, C). Therefore, our observations in these transient analyses suggested that *PfCNR1* downregulation specifically increased the berry size in *Physalis* species. We further analysed the consequence of the stable ‘altered *PfCNR1* expressions’ in transgenic *P. floridana* plants.

### *PfCNR1* negatively controls cell proliferation and thus organ size in *P. floridana*

**Fig. 4.**
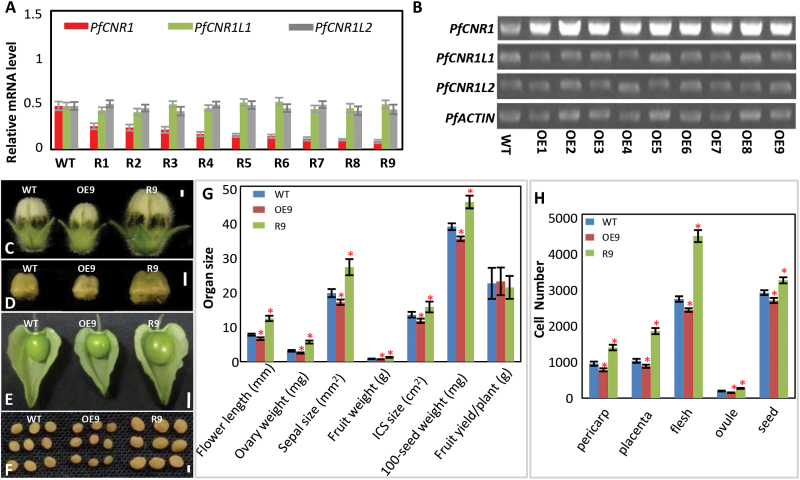
Altering *PfCNR1* expression in *P. floridana* using transgenic approaches. (A) Genotyping *PfCNR1* RNAi transgenic plants. The expression of the three genes (*PfCNR1*, *PfCNR1L1*, and *PfCNR1L2*) in the *PfCNR1* family was investigated among the wild type (WT) and nine lines of *35S:PfCNR1*-RNAi designated R1–R9. *PfACTIN* was used as an internal control. Three independent biological samples of each line were subjected to qRT-PCR. The mean and standard deviation are presented. (B) Genotyping of *PfCNR1* overexpression transgenic plants. The expression of three genes (*PfCNR1*, *PfCNR1L1*, and *PfCNR1L2*) in the *PfCNR1* family was investigated among the wild type (WT) and nine lines of *35S:PfCNR1*, designated OE1–OE9. *PfACTIN* was used as a loading control. Three independent biological samples of each line were subjected to RT-PCR, and a typical gel image is presented. (C–F) Comparison of organs among wild type (WT), *35S:PfCNR1* (OE9), and *35S:PfCNR1*-RNAi (R9). (C) flowers of the wild-type (WT), *PfCNR1* overexpressor (OE9) and RNAi plant (R9). Bar 1mm. (D) Carpels of WT, OE9, and R9. Bar, 1mm. (E) Mature fruit of WT, OE9, and R9. Bar, 1cm. (F) Mature seed of WT, OE9, and R9. Bar, 1mm. (G) Quantification of organ size variation among the WT, OE9 and R9. Three plants for each line were cultivated. Nine mature flowers from each line and the ovaries and sepals from these flowers were measured. Berry weight, ICS size and 100-seed weight were recorded from 10 fruits per plant. The total berry yield for each plant was measured. In each measurement, the mean and the standard deviation are presented. (H) Cell number variation among the WT, OE9 and R9. The details were presented in Table S8. The significance compared with WT was evaluated using a two-tailed *t*-test. A star indicates a significant difference (*P*<0.05).

We created nine lines of transgenic *P. floridana* from independent explants where *PfCNR1* expression was downregulated via RNAi (Fig. 4A). Multiple organ sizes, including leaves, floral organs, berries, and seeds, were significantly increased in comparison with the wild type (Fig. 4C–G; Supplementary Fig. S5 and Supplementary Table S7 at *JXB* online). A negative correlation was found between *PfCNR1* transcript levels and organ sizes; nonetheless, seed number per berry, total fruit yield, and plant weight (fresh weight of the above-ground plant at harvest) were indistinguishable (*P*>0.05) between transgenic and wild-type plants (Supplementary Table S7). Thus, *PfCNR1* is a negative regulator of organ size (weight) without altering the total biomass or yield.

Organ size alteration may result from alterations in cell size and/or cell number. We found that the size of the epidermal cells of leaves, calyces, and ICSs in the *35S:PfCNR1*-RNAi transgenic lines was not altered in comparison with the wild type (Supplementary Table S8 at *JXB* online), which was corroborated by an unchanged cell number per unit area (Supplementary Fig. S5B, C, G). In contrast, cell number was significantly increased but no significant difference in cell size was observed in the ovaries, developing berries, and seeds between the RNAi and wild-type plants (Fig. 4H; Supplementary Fig. S5D, E, and Supplementary Table S8). In mature berries of *35S:PfCNR1*-RNAi transgenic plants (i.e. R9), the cell size decreased as the cell number increased compared with the wild type (Supplementary Table S8). This can be attributed to the compensation effect between cell number and cell size that has been reported previously (Doonan, 2000).

The findings were further substantiated by overexpressing *PfCNR1* in *P. floridana*. Nine independent transgenic lines were generated from independent explants (Fig. 4B). Most of the nine transgenic plants showed a significant reduction in multiple organ sizes; i.e. in leaves, flowers, ICSs, berries, and seeds (Fig. 4C–G; Supplementary Fig. S5 and Supplementary Table S7). Nonetheless, seed number per berry, berry yield per plant, and plant weight were not significantly altered (*P*>0.05) in these transgenic plants overexpressing *PfCNR1* (Supplementary Table S7). Cell number was reduced significantly but no significant changes (*P*>0.05) in the cell size of organs were seen in the transgenic plants (Fig. 4H; Supplementary Fig. S5B–E, G and Supplementary Table S8). In comparison with the wild-type *P. floridana* plants, the siblings that segregated from the T_2_ populations of some transgenic lines did not show any phenotypic variation, and the *PfCNR1* expression was also not altered in these plants (Supplementary Fig. S5H–J). Thus, *PfCNR1* controls organ size by inhibiting cell division.

### PfCNR1 is a plasma membrane-anchored protein

To understand how *PfCNR1* exerts its function, we next determined the subcellular localization of its encoded protein. In subcellular localization analyses, GFP alone was distributed in both the nucleus and cytoplasm (Supplementary Fig. S6A at *JXB* online). In marked contrast, the fused protein PfCNR1–GFP was localized exclusively at the periphery of the cell (Supplementary Fig. S6B, D), and the GFP signal did not surround the nuclei (Supplementary Fig. S6B, C), which suggests that no protein was anchored on the vacuolar or the nuclear membrane. When the plant cells were plasmolysed, PfCNR1–GFP was clearly demonstrated to be located in the plasma membrane regardless of the residual GFP signals adhering proximate to the cell wall (Supplementary Fig. S6D, E). This suggested the unique localization of PfCNR1 in the plasma membrane.

**Fig. 5.**
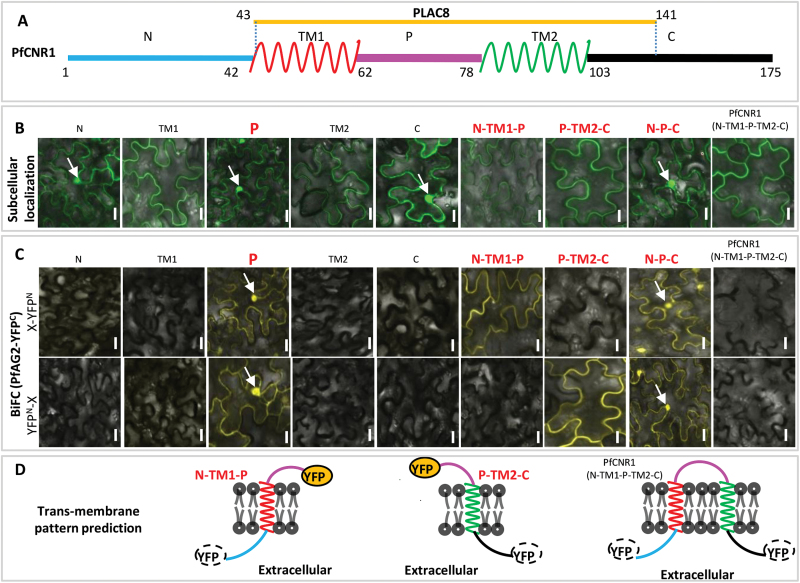
Interaction between PfAG2 and the membrane-anchored PfCNR1. (A) Subsections of PfCNR1 based on computational prediction. Different sections of the protein are highlighted in colour. N, N-terminal region; TM (TM1 and TM2), transmembrane domain; P, the portion between two transmembrane domains; C, C-terminal region. The number indicates the position of the amino acids of the two ends of each section. (B) Subcellular localization of each section of PfCNR1. The fusion protein of each section with GFP was transiently expressed in plant cells. (C) BiFC assays. PfAG2–YFPc and each section of PfCNR1 were co-expressed in plant cells. X indicates different sections of PfCNR1. The arrow indicates the nuclei. Bars, 10 μm. (D) Subcellular localization predication of each section of PfCNR1 and the mutated PfCNR1. The predication was performed according to the observations in (B) and (C). The dash-circled YFP indicates that the YFP signal was not seen when YFPn was fused at the extracellular end, while the solid-circled yellow YFP indicates that the YFP signal was detected once the YFPn was fused at the intracellular end. The colours of each PfCNR1 section are as used in (A).

Based on the computational prediction (Supplementary Fig. S6F), PfCNR1 was expected to consist of the N_1–42_, TM1_43–61_, P_62–78_, TM2_79–103_, and C_104–175_ portions (Fig. 5A). To elucidate further the cell membrane localization of PfCNR1, we made different constructs that synthesized the GFP fusion with each portion or portion combinations (e.g. N–P–C), respectively. In plant cells, all expressed constructs that did not contain any transmembrane portion (TM1 or TM2) generated a GFP signal in the nuclei; otherwise, no nuclear GFP signal was seen (Fig. 5B). Thus, PfCNR1 is very probably localized in the cell membrane. PfCNR1 also had the PLAC8 domain aa 43–141, which mainly covered TM1, P, and TM2 (Fig. 5A). PLAC8 is an FW2.2-like feature and this domain is suggested to be involved in transmembrane localization and protein–protein interaction (Libault and Stacey, 2010).

### Characterization of interacting proteins of PfCNR1

To exert its role in the cell cycle, we assumed that the cell membrane-anchored PfCNR1 should interact with other proteins, and then transduce the signal into the nucleus to modulate cell division. Therefore, we decided to search for PfCNR1 interacting proteins. PfCNR1 and its different portions (defined by clues from Fig. 5F) did not self-activate in yeast, so they were used as respective baits to screen a *Physalis* expressing library (He *et al.*, 2007). Sequencing analyses revealed that PfGIP (a homologue of gibberellin-induced protein in *P. floridana*), PfSEP1 [a SEPALLATA (SEP)-like MADS-domain protein in *P. floridana*], and PfAG2 [an AGAMOUS (AG)-like MADS-domain regulatory protein in *P. floridana*] probably interacted with PfCNR1 (Supplementary Fig. S7A at *JXB* online). They were either partial or full sequences in length; for example, five recombinant yeast colonies inserting the coding sequence of *PfAG2* were obtained, and two full-length sequences were seen (Supplementary Fig. S7A and B). To further confirm their interactions, we isolated the full-length sequences of all these putative partners, and the interactions were then tested in yeast via co-transformation (see Materials and methods). We found that interaction of PfCNR1 and PfAG2 was robust in transformed yeast cells; however, the interaction was not revealed in BiFC assays in plant cells (Supplementary Fig. S7C, F). PfAG2 was a homologue of the homeotic MADS-domain protein AG in *Arabidopsis* (Supplementary Fig. S7G). We further examined the dimerization of PfCNR1, and found that PfCNR1 and its different portions did not form dimers in yeast and plant cells (Supplementary Fig. S7F). The putative orthologue of the casein kinase II β-subunit 1 (CKIIβ1) is a known interacting partner of tomato FW2.2 (Cong and Tanksley, 2006); therefore, we also isolated the putative orthologue *P. floridana PfCKIIβ1* and included it in our protein–protein interaction studies (Supplementary Fig. S7H). PfCNR1 was found to interact with PfCKIIβ1 in yeast as well; however, no YFP signal was detected in the plant cells under any of the circumstances tested (Supplementary Fig. S7F). In BiFC assays, YFPn was fused to both the N and C termini of PfCNR1-derived constructs and each of them was co-expressed with the fusion protein PfAG2 or PfCKIIβ1 with YFPc in plant cells. In principle, the YFP signal should be reconstituted once the two proteins interact. The failure to detect a YFP signal may have been because of the non-overlapping localization of PfCNR1 and its putative interacting proteins. To clarify this, subcellular localization of these putative partners was checked, and they were localized in either the nuclei or the cytoplasm (Supplementary Fig. S7F). Thus, it is likely that the two ends (N- and C-terminal ends) of PfCNR1 might be extracellular, as suggested by bioinformatics prediction (Fig. 5F). To confirm this experimentally, we used different portions of PfCNR1 to check the interactions with the above-mentioned PfCNR1 partners using BiFC assays. The YFP signal was only observed when the intracellular *PfCNR1*_62–78_ encountered either PfAG2 or PfCKIIβ1 in plant cells (Supplementary Fig. S7C–F), thus corroborating our assumption regarding *PfCNR1* transmembrane pattern. Moreover, the *PfCNR1*_62–78_ portion was required for interacting with its partners PfAG2 and PfCKIIβ1.

To the best of our knowledge, this is the first reported finding of an FW2.2-like protein interacting with an AG-like MADS-domain protein, and thus the interaction of PfCNR1 and PfAG2 was analysed further. YFPn was fused to both the N and C termini of PfCNR1-derived constructs (Fig. 5A) and each was co-expressed with PfAG2–YFPc in plant cells. The YFP signal was only detected once the P portion of PfCNR1 was included; moreover, the presence of YFP was dependent on the position of the YFPn fusion if any one of the transmembrane domains TM1 and/or TM2 was included (Fig. 5C). These results further suggested that the 16 aa peptide of PfCNR1 is required for its interaction with PfAG2 and also suggested the cell membrane localization pattern of PfCNR1and its derived proteins (Fig. 5D).

### Transcriptional correlation of *PfCNR1* and *PfCYCD2;1*

**Fig. 6.**
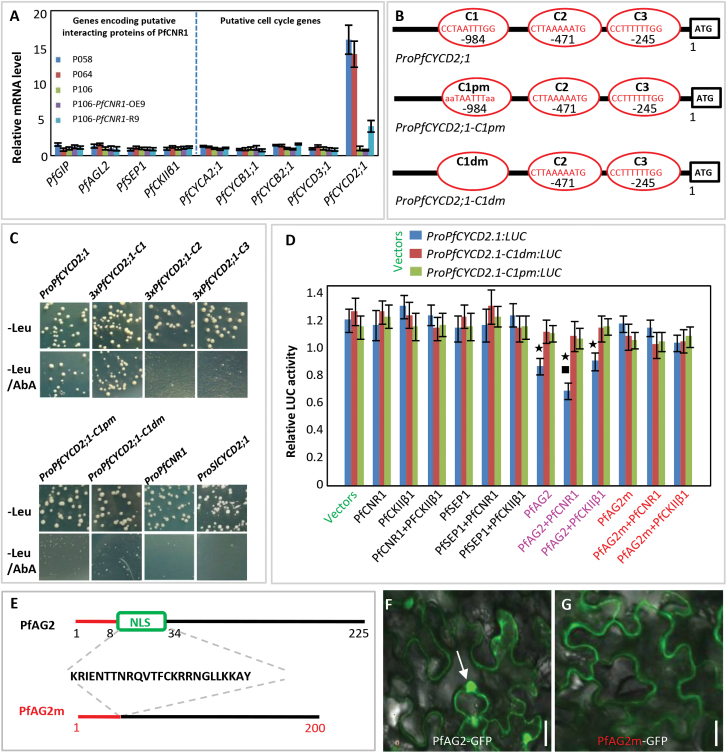
Regulation of the *PfCYCD2;1* expression. (A) Gene expression analysis of the putative PfCNR1 interacting partners and the putative *Cyclin*. Total RNA from the B2 flower buds was subjected to qRT-PCR. *PfACTIN* was used as an internal control. Expression of each gene was investigated in three independent biological samples and its expression in the wild-type P106 was set as 1. The mean and standard deviation are presented. (B) Characterization of the putative *PfCYCD2;1* promoter. Three CArG-boxes (C1, C2, and C3) are highlighted in red circles beyond the ATG. The mutated promoters resulting from either a point mutation (C1pm) or deletion mutation (C1dm) are shown. The putative promoter sequences are presented in Supplementary Fig. S8. (C) Yeast one-hybrid assays between PfAG2 and the related DNA fragments. Pro, promoter; 3×, three tandem repeats of the CArG-box; C1, CArG-box1; C2, CArG-box2; C3, CArG-box3; dm, deleted mutation; pm, point mutation. The survival of yeast cells on SD/–Leumedium supplemented with aureobasidin A (AbA) suggested that the protein could bind to the corresponding DNA fragment. (D) LUC relative activity assay in *P. floridana* protoplasts. LUC activities were normalized using *35S:GUS* as an internal control. The wild-type PfAG2 associated interactions are highlighted in pink and the interactions of PfAG2m that excluded the NLS are indicated in red. The mean and standard deviation from at least six independently repeated assays are presented. The star and the square, respectively, indicate a significant difference (*P*<0.05) compared with *ProPfCYCD2;1:LUC* or *ProPfCYCD2;1:LUC* with PfAG2. (E) The NLS in PfAG2 and its deleted version (PfAG2m). The 25 aa sequences of the NLS are given between the PfAG2 and PfAG2m structures. (F) Subcellular localization of PfAG2. The arrow indicates the nuclei. (G) Subcellular localization of PfAG2m. Bar, 10 μm (F, G).

Transcriptional regulation by the *PfCNR1* signalling pathway were also investigated. The expression of the putative *PfCNR1* interacting partner genes was first checked, and they were all expressed but no differential expression in the B2 flower buds was observed among P058, P064, P106, *35S: PfCNR1* (OE9), and *35S: PfCNR1*-RNAi (R9) plants (Fig. 6A). Since cell division in eukaryotic organisms is controlled by highly conserved basic cell-cycle machinery (Dewitte and Murray, 2003), we identified five putative *Cyclin* genes from *P. floridana* that are key genes in the plant cell division cycle (Mizukami, 2001). We designated these genes *PfCYCA2;1*, *PfCYCB1;1*, *PfCYCB2;1*, *PfCYCD2;1*, and *PfCYCD3;1*. In the B2 flower buds of P058, P064, R9, and OE9 plants, only *PfCYCD2;1* was significantly elevated in P058, P064, and R9 plants, and it was slightly repressed in OE9 plants relative to the wild-type P106 (Fig. 6A). The expression of *CyclinD2;1* positively correlated with cell division (Oh *et al.*, 2008; Talengera *et al.*, 2012). Therefore, *PfCNR1* may function as a negative modulator of the cell division cycle by directly or indirectly influencing the expression of *PfCYCD2;1*.

### Molecular interactions of the PfCNR1 interacting proteins and *PfCYCD2;1*

In order to elucidate the molecular interactions of *PfCYCD2;1* with the PfCNR1 protein and its interacting partners, we first checked the protein-protein interactions among these proteins. We found no evidence to support any possible reciprocal direct contact among PfAG2, PfCKIIβ1 and PfCYCD2;1 (Supplementary Fig. S7I). Moreover, PfCNR1 did not interact with PfCYCD2;1 either (Supplementary Fig. S7I).

Since PfAG2 is a putative MADS-domain transcription factor (Supplementary Fig. S7G), its molecular interaction with the *PfCYCD2;1* gene is of potential interest. To reveal the molecular interaction, we isolated the 1.7kb *PfCYCD2;1* promoter. We found three CArG-boxes (C1, C2, and C3) in this region (Fig. 6B; Supplementary Fig. S8 at *JXB* online). Interestingly, only one CArG-box was found in the orthologous region in tomato (Supplementary Fig. S8). The CArG-box is a well-known binding site for the MADS-domain transcription factors (Kaufmann *et al.*, 2005). In yeast one-hybrid assays, the survival of yeast cells on SD/–Leu medium supplemented with AbA suggested that the protein could bind to the DNA fragment. We therefore found that PfAG2 could bind to the *PfCYCD2;1* promoter but not to the *SlCYCD2;1* and *PfCNR1* promoters (Fig. 6C). In particular, PfAG2 could specifically interact with the C1 CArG-box. Once the CArG-box was deleted or mutated, PfAG2 did not bind to the mutated *PfCYCD2;1* promoters. PfSEP1, another MADS-box protein, did not bind to the promoter of *PfCYCD2;1*, *SlCYCD2;1*, or *PfCNR1*, nor did it bind to any CArG-box in the *PfCYCD2;1* promoter (Supplementary Fig. S9 at *JXB* online).

We next substantiated the consequence of the specific binding. We exploited this in *P. floridana* protoplasts using the LUC gene as a reporter (Fig. 6D). We found that PfAG2 could significantly (*P*<0.05) repress LUC expression driven by the *PfCYCD2;1* promoter and co-expression of PfCNR1 and PfAG2 could further inhibit LUC expression (*P*<0.05). Furthermore, when the C1 CArG-box in the *PfCYCD2;1* promoter was mutated or deleted, the repression of LUC expression by PfAG2 and PfCNR1 disappeared. Nonetheless, altering *PfCNR1* expression in the leaf protoplasts alone did not affect the expression of the reporter gene (Fig. 6D; Supplementary Fig. S10A at *JXB* online). Furthermore, altering expression of *PfCKIIβ1* or *PfSEP1*, and co-expression of *PfCNR1* with either *PfCKIIβ1* or *PfSEP1*, also did not affect LUC expression driven by the *PfCYCD2;1* promoter either (Fig. 6D). Nevertheless, *PfAG2* mRNA was not detected in the protoplasts from young leaves (Supplementary Fig. S10B). These data therefore suggested that PfAG2 could directly and specifically repress *PfCYCD2;1* expression.

The interaction between PfCNR1 and PfAG2 was able to suppress LUC expression driven by the *PfCYCD2;1* promoter, and the nuclear import of PfAG2 seems to be essential. The NLS of PfAG2 was deleted to produce PfAG2m (Fig. 6E). Unlike PfAG2, PfAG2m could not be imported into the nucleus (Fig. 6F, G). In transient LUC assays, LUC expression driven by the *PfCYCD2;1* promoter was no longer suppressed once the PfAG2m was expressed instead of PfAG2 (Fig. 6D). Therefore, the nuclear import of PfAG2 is essential to suppress *PfCYCD2;1* expression, thereby regulating cell division and organ size. Moreover, we found that among *Physalis* species, *PfCNR1* transcript levels at B2 flower buds were negatively correlated with *PFCYCD2;1* mRNA levels (*r*=–0.50, *P*=0.04), which were positively correlated with organ size (Supplementary Table S6).

## Discussion

Fruit size is a prime target in the breeding of Solanaceous crops. As the first cloned QTL, *FW2.2* accounts for approximately 30% of the fruit size variation between cultivated tomato and its wild-type relatives (Frary *et al.*, 2000). Its homologues are all associated with organ size or cell division in maize, rice, avocado, cherry, soybean, and melon (Dahan *et al.*, 2010; Guo *et al.*, 2010; Libault *et al.*, 2010; Guo and Simmons, 2011; Franceschi *et al.*, 2013; Xu *et al.*, 2013; Monforte *et al.*, 2014); thus, they were also named *Cell Number Regulator* (*CNR*) genes. Nonetheless, how they regulate cell division is not understood. In the present study, we characterized *FW2.2*-like genes in *P. floridana*, also termed *PfCNR1*-like, and suggested a novel regulatory pathway of *PfCNR1* (characterized as the putative *FW2.2* orthologue) in cell division, and found that the heterochronic expression of the *PfCNR1* genes in the ovaries might explain interspecific variation in berry size and seed weight within *Physalis* species.

### A novel working model for membrane-anchored PfCNR1 in the cell cycle

**Fig. 7.**
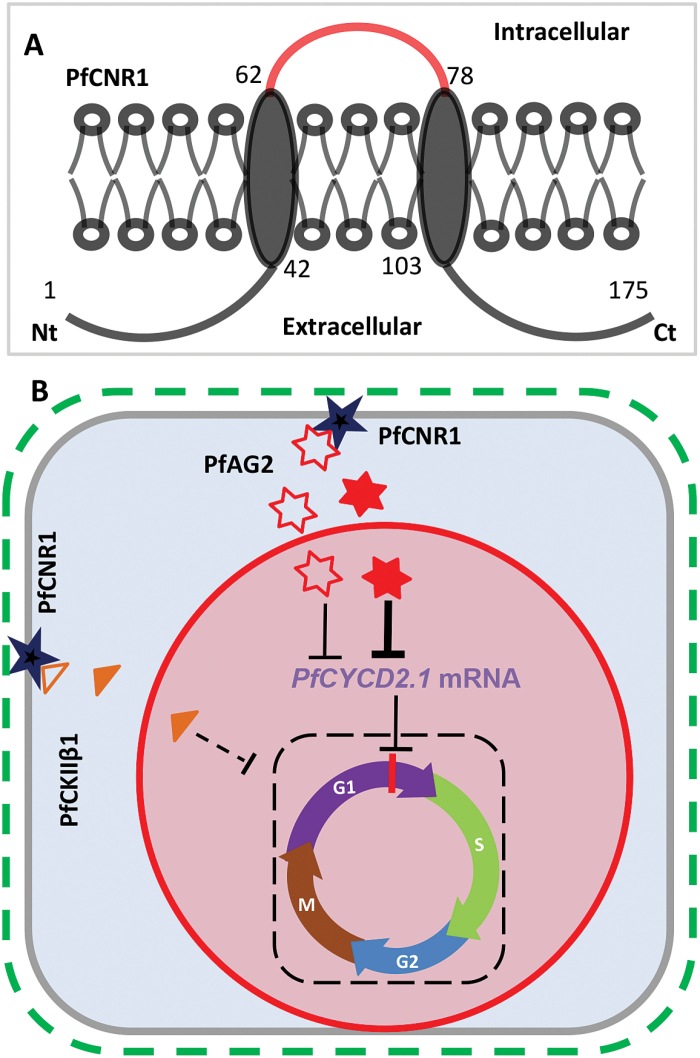
PfCNR1-related models. (A) PfCNR1 membrane localization pattern. Both the N-terminal (Nt) and C-terminal (Ct) regions are extracellular. The intracellular portion (P) of PfCNR1 that mediated interaction with PfAG2 is highlighted in red. (B) A novel working model of PfCNR1 regulating cell cycle. The proposed PfCNR1 activity in the cell cycle is shown in a stylized cell. The green dashed box indicates the cell wall. The grey box indicates the cell membrane. The red circle indicates the nuclear membrane and the black dashed box inside depicts the four phases of the cell cycle (G_1_, S, G_2_, and M). The red line on the G_1_ phase indicates a check point of the cell cycle. The black star on the cell membrane is PfCNR1. The outlined triangle is PfCKIIβ1, and the yellow-filled triangle represents PfCKIIβ1, which is putatively modified by interacting with PfCNR1. The red outlined star represents PfAG2 and the red-filled star is PfAG2, which is putatively modified by interacting with PfCNR1. The PfCKIIβ1- and PfAG2-mediated pathways seem to be independent since PfCKIIβ1 did nor interact with PfAG2 or replace PfAG2. The black T-line indicates the repression effect of the upstream genes. PfAG2 represses *PfCYCD2;1* expression, and the modified PfAG2 after interacting with PfCNR1 enhances the repression. The interacting and regulatory pathway of PfCNR1–PfAG2–*PfCYCD2;1* for cell division established in the present work is indicated by solid T-lines while the dashed T-line indicates the repression effect of the PfCNR1–PfCKIIβ1 pathway that needs to be substantiated.

Similar to PfCNR1, FW2.2 and its homologous proteins in soybean and rice are suggested to be localized to the plasma membrane (Xu *et al.*, 2013; Cong and Tanksley, 2006; Libault *et al.*, 2010). These proteins were proposed to have one to two transmembrane domains in the PLAC8 domain, and the variation of the number of transmembrane domains between these proteins may lead to a different organization of the core domain relative to the membrane, and thus they may help mediate protein–protein interactions or be involved in the cellular signalling process (Libault and Stacey, 2010). In our work, based on computational and experimental approaches, we have proposed a detailed model for PfCNR1 subcellular localization (Fig. 7A). Both the N and C termini are extracellular and only the middle 16 aa section (P section) is intracellular. However, the fact that these CNR/FW2.2 proteins regulate cell division is intriguing. They may facilitate the transport of ions such as cadmium and calcium across membranes, but how regulation of ion transport would lead to changes in cell division is unknown (Song *et al.*, 2004; Nakagawa *et al.*, 2007; van der Knaap *et al.*, 2014). We assumed that the FW2.2/CNR proteins need to interact with other proteins for signal transduction to direct cell division in the nuclei. A large portion of the protein is extracellular, which could perceive the external signal for cell division control, while the intracellular portion could mediate the interaction with proper protein partners. For example, tomato FW2.2 interacts with CKIIβ1 (Cong and Tanksley, 2006). CKIIβ1 plays a critical role in cell growth and proliferation in yeast (Roussou and Draetta, 1994), thereby suggesting an unconfirmed CKIIβ1-mediated model. The P section determined in PfCNR1 may mediate its interaction with specific interacting partners and thus determine its biological functionality. The CKIIβ1-mediated cell division pathway might be present in *Physalis* species, since PfCNR1 interacted with PfCKIIβ1 in *P. floridana*. We also found that PfCNR1 interacted with the MADS-domain protein PfAG2 and that PfAG2 could bind to a CArG-box in the *PfCYCD2;1* promoter and repress the *PfCYCD2;1* expression. Therefore, the expression of *PfCYCD2;1*, a key gene for the G_1_/S transition phase in the cell cycle (Mizukami, 2001; Oh *et al.*, 2008; Talengera *et al.*, 2012), was negatively correlated with *PfCNR1* expression levels. In this regulatory pathway, binding the *PfCYCD2;1* promoter by PfAG2 in the nuclei is essential. The interaction of PfCNR1 with PfAG2 is not required, but it enhances the repressive activity on *PfCYCD2;1* expression. The intracellular 16 aa peptide is required for the interaction of PfCNR1 and PfAG2. This PfAG2-mediated regulation of *PfCYCD2;1* represents a novel alternative regulatory pathway for the PfCNR1-like proteins integrated into the cell cycle. The proposed pathway seems to be independent of the PfCKIIβ1-mediated pathway, since PfAG2 did not interact with PfCKIIβ1, and since manipulating *PfCKIIβ1* expression did not affect LUC expression driven by the *PfCYCD2;1* promoter. In both regulatory pathways, certain biochemical modifications may be ascribed to PfAG2 or PfCKIIβ1 through their interactions with PfCNR1, which requires further investigation. Nonetheless, our work enriches the details of the PfAG2-mediated working model for PfCNR1-directed cell division (Fig. 7B). The MADS-domain proteins are major determinants of floral organ identity, and a molecular link between floral organ identity and growth is missing (Dornelas *et al.*, 2011). *PfAG2* encodes a closely related homologue of the *Arabidopsis* homeotic C-function transcription factor AG, which specifies carpel identity (Yanofsky *et al.*, 1990). Thus, our work may provide a first glimpse at the molecular link between ovary identity and growth. To what extent the CNR–AG–*Cyclin* pathway is conserved in angiosperms is not yet clear. Expansion of the related gene family with subsequent divergence during evolution might complicate the situation, but comparative and *in planta* functional analyses of these molecular interactions among the *FW2.2*-like genes, the *AG*-like genes, and the *Cyclin*-like genes will clarify this. Since ectopically overexpressing the *PfCNR1*-like genes could affect multiple organ sizes, as in maize (Guo *et al.*, 2010) and *P. floridana*, our work suggests a working model for a cell membrane-anchored protein that modulates cell division and thereby governs organ size determination in plants.

### Altering expression is essential in PfCNR1-like function recruitment

*GmFWL1* is uniquely expressed in roots and nodules, and affects nodule organogenesis (Libault *et al.*, 2010). However, expression specification of *FW2.2*-like genes often is associated with organ size variation in various plant species including tomato, maize, rice, cherry, avocado, and melon (Frary *et al.*, 2000; Dahan *et al.*, 2010; Guo *et al.*, 2010; Guo and Simmons, 2011; Franceschi *et al.*, 2013; Xu *et al.*, 2013; Monforte *et al.*, 2014). Interestingly, *FW2.2* is expressed early in floral development and controls carpel cell number (Frary *et al.*, 2000). This gene negatively regulates tomato fruit size via allelic variation in gene expression rather than variation in the FW2.2 protein sequences (Cong *et al.*, 2002; Liu *et al.*, 2003). Expression of the larger fruit allele has an early and short duration, whereas expression from the smaller fruit allele peaks later and persists for a long period. Therefore, the heterochronic expression of *FW2.2* is involved in tomato domestication (Cong *et al.*, 2002). *PfCNR1* was highly expressed in ovaries, ovules, and floral calyces, controlling multiple post-floral organ sizes including berries, seeds, and ICSs. Moreover, *PfCNR1* transcript levels in the ovaries were negatively correlated with *PfCYCD2;1* expression, mature fruit weight, and 100-seed weight among the *Physalis* species. This suggests a recruitment of the PfCNR1–PfAG2–*PfCYCD2;1* function during the evolution of fruit sizes within *Physalis* species. Unlike *FW2.2* (Frary *et al.*, 2000; Cong *et al.*, 2002), expression of the smaller berry *PfCNR1* allele peaked earlier and was higher than that of the larger berry allele, and hence both heterochronic expression and differential mRNA levels of the *PfCNR1* alleles may contribute to the evolution and development of berry size in *Physalis* species.

In the domestication of tomato fruit size, *FW2.2* accounts for the ﬁrst key step, while increasing the carpel number by *FAS* and *LC* is considered the second step (Cong *et al.*, 2008; Muños *et al.*, 2011). QTL analyses have revealed that Solanaceous crops might share a common genetic basis for domestication as in tomato, eggplant, and pepper (Doganlar *et al.*, 2002; Paran and van der Knaap, 2007; Wang *et al.*, 2008). No differences were observed in the carpel number (two locules) in *Physalis* species; thus, *Physalis* domestication might be different from tomato. Nonetheless, recruiting a PfCNR1-like function for a different cell number in the ovary might be the first crucial step in the evolution of different species with a different fruit size as an adaption for seed dispersal. Besides, human selection might act on the particular species for the berry yields. *P. philadelphica* (tomatillo) is a domesticated species (Montes Hernández and Aguirre Rivera, 1994), and the berry size varied from about 1.2 to 11.2g (Wang *et al.*, 2012). The cell number in ovaries, a consequence of recruiting a PfCNR1 function, seemed to be comparable among tomatillo accessions. However, the berry size variation in the species might correlate with differences in cell expansion of the developing berries, which would suggest an involvement of cell expansion regulators in determining tomatillo berry size. Very recently, *Physalis Organ Size1* (*POS1*) encoding a putative regulatory proteins with double cytokinin response factor (CRF)– APETALA2 (AP2) domains was found to act as a promoter of cell expansion, and expression variation of this gene correlates to natural variation of berry size in *P. philadelphica* (Wang *et al.*, 2014). Further studies can investigate the genetic interaction of the *PfCNR1* and *POS1* genes in the control of berry size in *Physalis* species.

## Supplementary data

Supplementary data are available at *JXB* online.

Supplementary Fig. S1. Median transverse sections of ovaries and berries.

Supplementary Fig. S2. A neighbour-joining (NJ) tree of *FW2.2*-related genes.

Supplementary Fig. S3. Expression of *PfCNR1-like* genes during flower and fruit development.

Supplementary Fig. S4. VIGS of the *PfCNR1* gene family in *Physalis floridana.*

Supplementary Fig. S5. Transgenic analyses of *PfCNR1* in *Physalis floridana*.

Supplementary Fig. S6. Transient PfCNR1 expression in plant cells.

Supplementary Fig. S7. Characterizations of the putative PfCNR1-interacting proteins.

Supplementary Fig. S8. Promoter alignment of *SlCYCD2;1* and *PfCYCD2;1*.

Supplementary Fig. S9. Yeast one-hybrid assays between PfSEP1 and the indicated DNA fragments.

Supplementary Fig. S10. LUC expression driven by the *PfCYCD2;1* promoter in *Physalis* leaf protoplasts.

Supplementary Table S1. *Physalis* resources used in the present work.

Supplementary Table S2. Primers used in the present work.

Supplementary Table S3. Variation in cell number in the ovaries and berries during development.

Supplementary Table S4. Variation of cell size in the ovaries and the berries during development.

Supplementary Table S5. Correlation between *PfCNR1*-like expression and ovary cell activities.

Supplementary Table S6. Variation in sequences and expressions, and their correlations with organ size within *Physalis* species.

Supplementary Table S7. Phenotypic variation of *35S:PfCNR1*-RNAi and *35S:PfCNR1* transgenic *Physalis* plants.

Supplementary Table S8. Cells in *35S:PfCNR1*-RNAi and *35S:PfCNR1* transgenic *Physalis* plants.

Supplementary Data

## Abbreviations:

BiFC: bimolecular fluorescence complementation
cDNA: complementary DNA
CNR: cell number regulator
GFP: green fluorescence protein
GUS: β-glucuronidase
LUC: luciferase
ORF: open reading frame
RNAi: RNA interference
TRV: tobacco rattle virus
VIGS: virus-induced gene silencing
YFP: yellow fluorescence protein.
