# Supplementary material for: Physalis floridana Cell Number Regulator1 encodes a cell membrane-anchored modulator of cell cycle and negatively controls fruit size
Source: J Exp Bot. 2014 Oct 11;66(1):257–70. doi: 10.1093/jxb/eru415 (PMC4265161; doi:10.1093/jxb/eru415)
Supplement: Supplementary Data [file supp_66_1_257__index.html]

 Physalis floridana Cell Number Regulator1 encodes a cell membrane-anchored modulator of cell cycle and negatively controls fruit size — Physalis floridana Cell Number Regulator1 encodes a cell membrane-anchored modulator of cell cycle and negatively controls fruit size — Supplementary Data 

# *Physalis floridana Cell Number Regulator1* encodes a cell membrane-anchored modulator of cell cycle and negatively controls fruit size

## Supplementary Data

Data files

**Files in this Data Supplement:**

- Supplementary Data - Supplementary Data
