# Supplementary material for: Physalis floridana Cell Number Regulator1 encodes a cell membrane-anchored modulator of cell cycle and negatively controls fruit size
Source: J Exp Bot. 2014 Oct 11;66(1):257–70. doi: 10.1093/jxb/eru415 (PMC4265161; doi:10.1093/jxb/eru415)
Supplement: Supplementary Data [file supp_eru415_jexbot132209_file001.pdf]

Journal of Experimental Botany

***Physalis floridana Cell Number Regulator1* encodes a cell membrane-anchored modulator of cell cycle and negatively controls fruit size**

Zhichao Li and Chaoying He

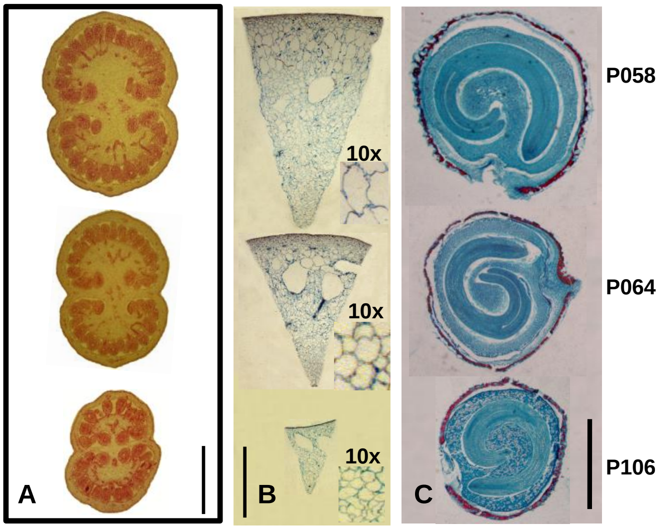

Figure S1. Median transverse sections of ovaries and berries.
(A) Median transverse sections of ovaries from mature flowers. (B) Median transverse sections of mature berries. 10 times enlarged sections are highlighted. (C) Transverse sections of mature seeds. From top to bottom: P058 (*P. philadelphica*), P064 (*P. philadelphica*) and P106 (*P. floridana*). Bars = 1 mm (A, C) and 0.5 cm (B).

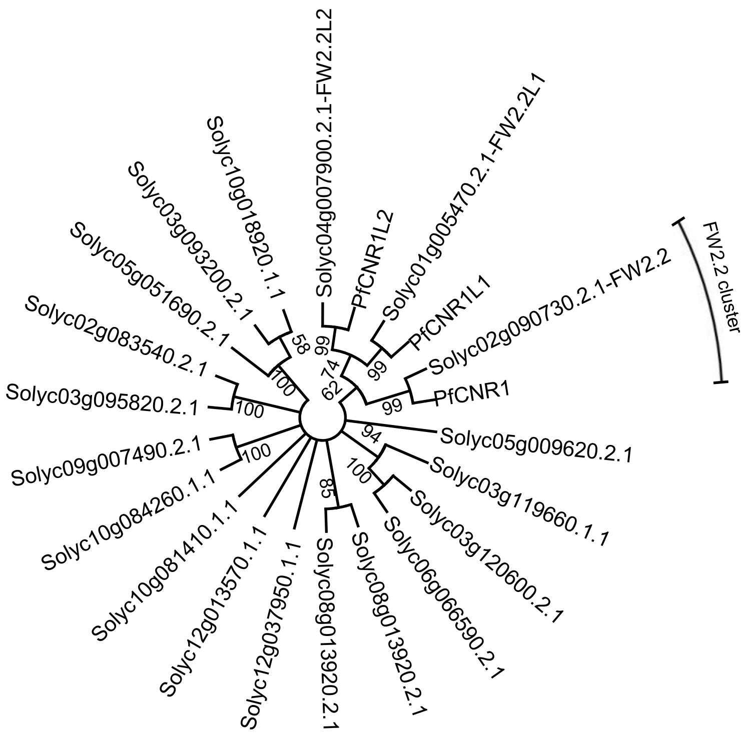

Figure S2. A neighbor-joining (NJ) tree of *FW2.2*-related genes. The sequences include tomato FW2.2 homologs (Franceschi et al., 2013) and *Physalis* PfCNR1 family (PfCNR1, PfCNR1L1 and PfCNR1L2). The bootstrap values (> 50) for the NJ method were shown.

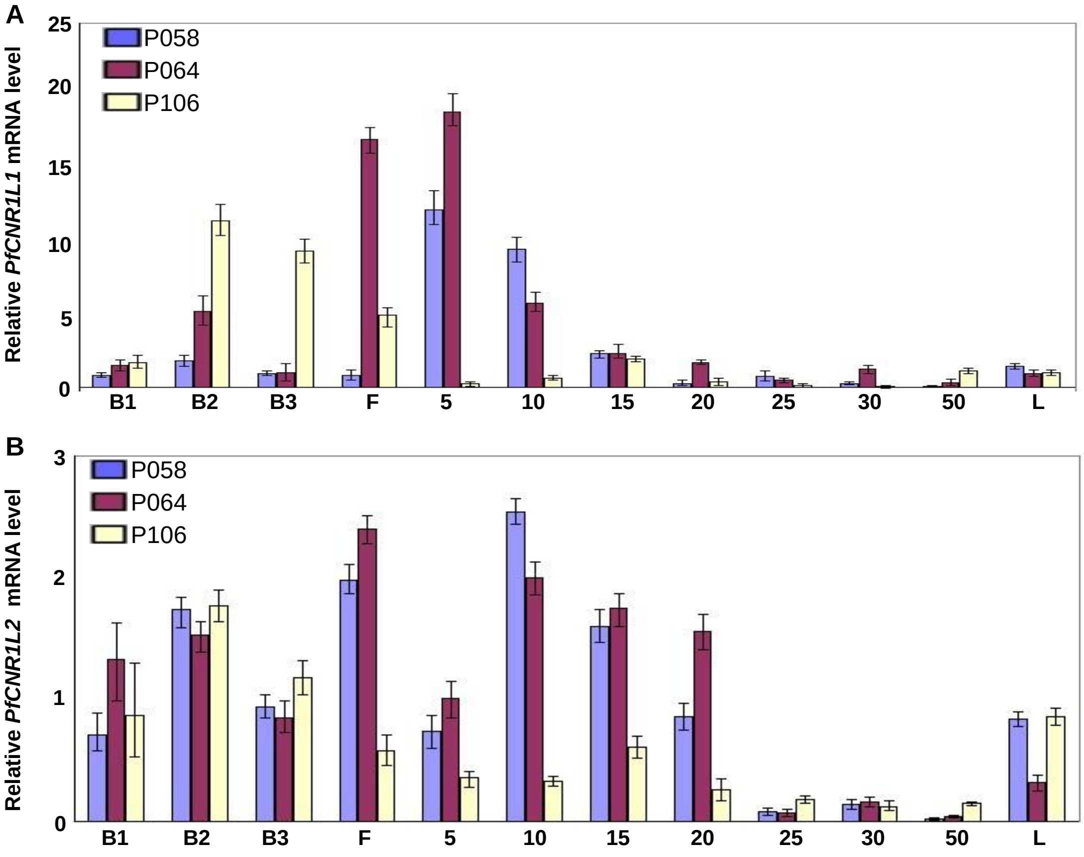

Figure S3. Expression of *PfCNR1-like* genes during flower and fruit development. (A) The *PfCNR1L1* expression. (B) The *PfCNR1L2* expression. Developmental stages are identical as defined in Figure 2A and B. L: leaf. *P. philadelphica* (P058), *P. philadelphica* (P064) and *P. floridana* (P106) were involved. *PfACTIN* was used as an internal control in qRT-PCR. The experiments were performed using three independent biological samples. The mean and the standard deviation are presented.

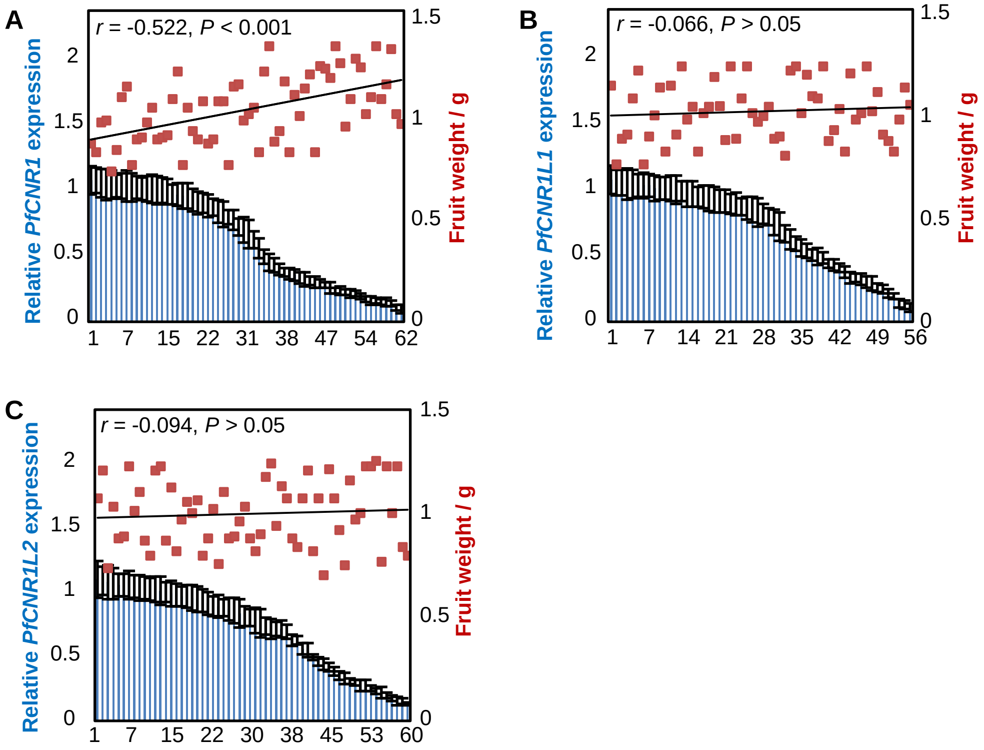

Figure S4. VIGS of the *PfCNR1* gene family in *Physalis floridana.*
(A) VIGS of *PfCNR1*. (B) VIGS of *PfCNR1L1.* (C) VIGS of *PfCNR1L2*. Total RNAs were isolated from 62 (A), 56 (B) and 60 (C) half-flowers for gene expression. Three technical repeats were performed using *PfACTIN* as an internal control. The mean and the standard deviation are presented. The fruits that developed from these half-flowers were weighed. In each case, correlations between gene expressions and fruit weights were evaluated which indicated that only *PfCNR1* from the family might control fruit size.

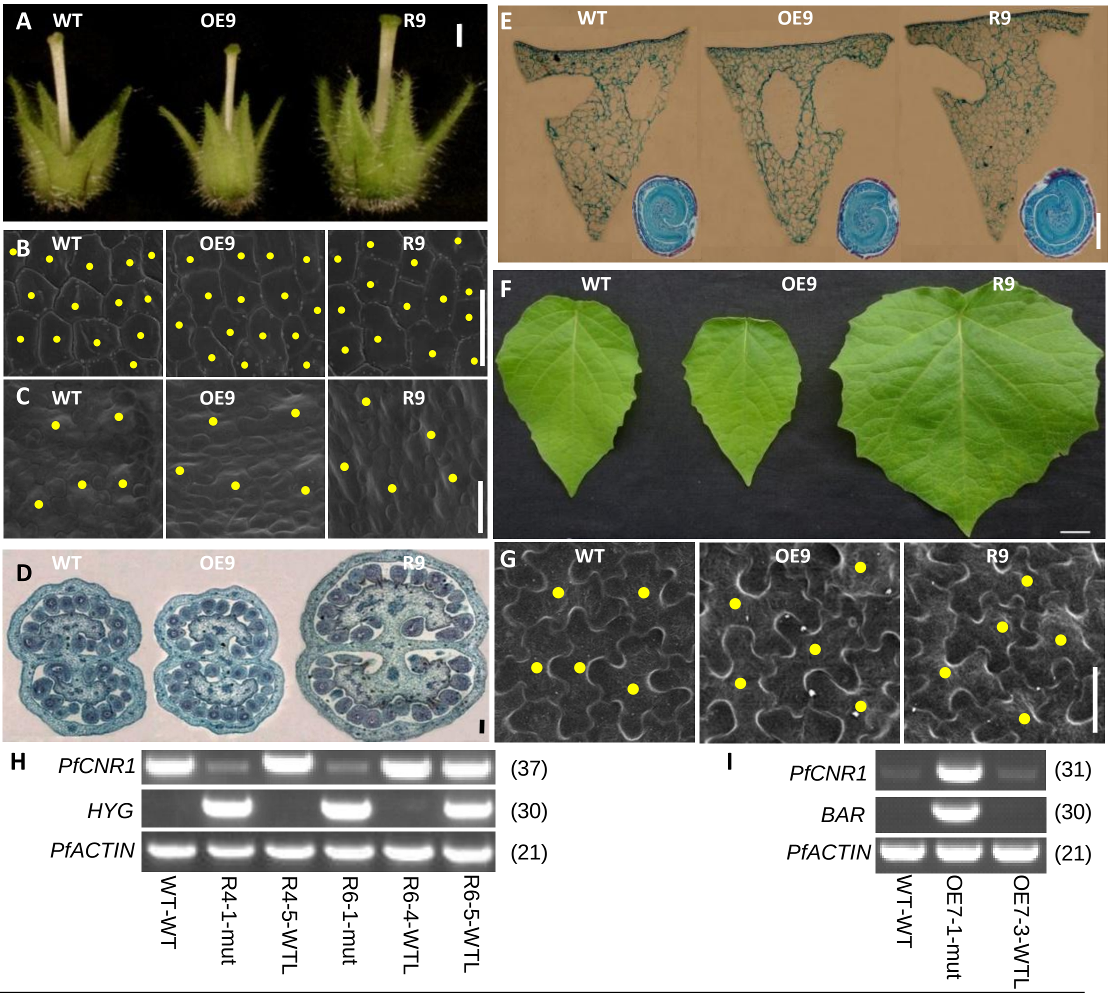

J

		Leaf size (mm^2)	Flower length (mm)	Carpel weight (mg)	Sepal size (mm^2)	Fruit weight (g)	ICS size (mm^2)	100-seed weight (mg)	Plant weight (g)	Fruit yield per plant (g)	Seed number per fruit
**WT**	WT	2320±210	8±0.7	3±0.4	20±1.9	0.8±0.1	1375±105	40±1	22.1±2.6	23.2±4.6	120±15
**RNAi**	R4-1-mut	2820±130**	10.8±0.5**	5.2±0.3**	26.8±1.7**	1.22±0.13**	1580±115**	44.5±1.3**	24.6	22.6	118±11
	R4-5-WTL	2280±130	8.3±0.5	2.8±0.3	21±1.5	0.85±0.1	1310±95	40.3±1.1	23.5	22.5	125±10
	R6-1-mut	2990±180**	11.4±0.7**	5.7±0.29**	27.8±1.8**	1.20±0.14**	1620±120*	45.8±1**	23.4	24.2	106±13
	R6-4-WTL	2370±170	7.8±0.7	3.1±0.5	20±1.5	0.82±0.13	1335±85	39.2±0.8	22.8	24.5	126±11
	R6-5-WTL	2220±120	8.2±0.5	3.2±0.4	19.3±1.2	0.8±0.1	1270±85	41.1±0.6	20.5	20.8	123±8
**OE**	OE7-1-mut	1550±60**	6.2±0.4**	2.3±0.12**	17.1±0.8**	0.62±0.05**	1190±80*	35.8±0.8**	23.8	20.3	114±10
	OE7-3-WTL	2450±180	8.3±0.5	3.2±0.4	18.9±1.7	0.7±0.1	1415±85	41.2±0.8	20.3	24.8	110±10

Figure S5. Transgenic analyses of *PfCNR1* in *Physalis floridana*.
(A-G) Comparison of organ and cell size between wild type (WT), *35S:PfCNR1* (OE9) and *35S:PfCNR1*-RNAi (R9). (A) Floral calyces and pistils. Bar = 1 mm. (B) Epidermal cells of mature flower calyces. Bar = 50 μm. (C) Epidermal cells of mature ICSs. Bar = 50 μm. (D) Paraffin sections of the ovaries from mature flower. Bar = 100 μm. (E) Paraffin sections of the mature berries and seeds. Bar = 1 mm. (F) Mature leaves. Bar = 1 cm. (G) Epidermal cells of the mature leaves. Bar = 50 μm. (H-J) Analyses of the siblings of some transgenic lines as indicated. (H) Genotyping analyses of the siblings segregated from the T_2 population of a few *PfCNR1* RNAi transgenic plants. The siblings with the wild-type-like (WTL) phenotype were observed in R4 and R6 lines, while a plant as indicated with the dramatic mutated phenotypic variation (mut) was included for comparison. These plants were also compared to the wild-type (WT). A transgenic plant (R6-5, the fifth plant segregated from the R6 line) that *PfCNR1* was not affected was included as well, but it showed the WTL phenotype. (I) Genotyping analyses of the T_2 population of *PfCNR1* overexpression transgenic line OE7. The total RNA from the flower buds of these plants was respectively subjected to RT-PCR. *HYG* is the hygromycin resistance gene in the pFGC-1008 vector. *BAR* is the phosphinothricin resistance gene in the pBAR vector. *PfACTIN* was used as an internal control. The number in the parenthesis is the amplification cycle number. (J) Phenotypic variation of a plant from the above analyzed *35S:PfCNR1*-RNAi and *35S:PfCNR1* transgenic *Physalis* lines. The differential significance of the organ size in transgenic plants compared to the wild-type organs was evaluated using two-tailed *t*-test. * indicates the significance at $P < 0.05$, and ** indicate the significance at $P < 0.01$.

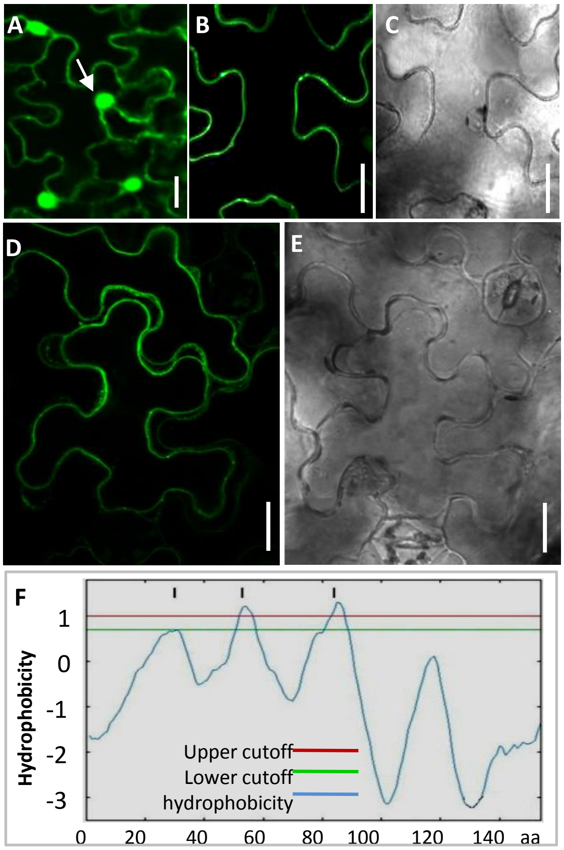

Figure S6. Transient PfCNR1 expression in plant cells. (A-E) Subcellular localization of PfCNR1 in tobacco leaf epidermal cells. Bars = 10 µm. (A) Super1300 vector (GFP alone). (B) PfCNR1-GFP. (C) Bright field of B. (D) PfCNR1-GFP in plasmolyzed cells. (E) Bright field of D. (F) Transmembrane domain of PfCNR1 was predicted using TopPred (http://www.sbc.su.se/~erikw/toppred2/).

A Prey Proteins	Baits: PfCNR1		
	1-42	103-175	1-175
PfGIP	0	0	2
PfAG2	0	1*	4
PfSEP1	3	0	0
PfCKIIβ1	0	0	0

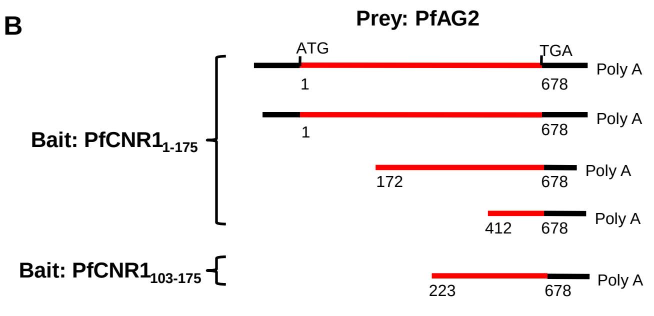

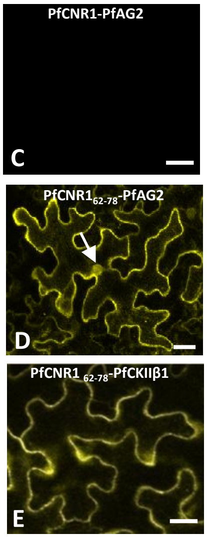

F Proteins	Yeast two-hybrid				BiFC		Subcellular localization
	PfCNR1$_{1-175}$ BK/AD	PfCNR1$_{1-42}$ BK/AD	PfCNR1$_{103-175}$ BK/AD	PfCNR1$_{62-78}$ BK/AD	PfCNR1 -YFP^N/ YFP^C	YFP^N / YFP^C -PfCNR1	GFP fusion
PfGIP	+ / +	− / −	− / −	− / −	− / −	− / −	Not analyzed
PfAG2	+ / +	− / −	− / −	+ / +	− / −	− / −	Cytoplasm, nucleus
PfSEP1	− / +	− / +	− / −	− / −	− / −	− / −	Nucleus
PfCKIIβ1	+ / +	+ / −	+ / +	+ / +	− / −	− / −	Cytoplasm, nu cleus
PfCNR1	− / −	− / −	− / −	− / −	− / −	− / −	Plasma membrane

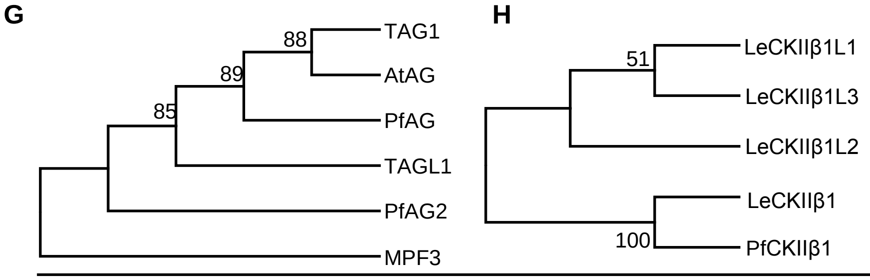

I	Yeast two-hybrid BK-AD/AD-BK	BiFC YFP^N-YFP^C / YFP^C-YFP^N
PfAG2−PfCKIIβ1	− / −	− / −
PfCYCD2;1−PfCNR1$_{1-175}$	− / −	− / −
PfCYCD2;1−PfCNR1$_{62-78}$	− / −	− / −
PfCYCD2;1−PfAG2	− / −	− / −
PfCYCD2;1−PfCKIIβ1	− / −	− / −

Figure S7. Characterizations of the putative PfCNR1 interacting proteins.
(A) The results of library screens using different versions of PfCNR1 as baits. The number indicates the independent colonies obtained. The colony that is highlighted with a star encodes a partial PfAG2. (B) The structures of the PfAG2 isolated in library screens. The four colonies encoding PfAG2 were obtained using PfCNR1 as baits include 2 full-length and 2 partial PfAG2. The number indicates the position of the sequences related to the first nucleotide of the open reading frame of the full length sequences of PfAG2. The coding region is highlighted using the red lines, while the untranslated region is indicated using the black lines. In the subsequent experiments, the PfAG2 cDNA that encodes a full-length protein was used without a special note. (C-E) BiFC assays of PfCNR1 and its interacting proteins. (C) PfCNR1-PfAG2. (D) PfCNR1$_{62-78}$-PfAG2. (E) PfCNR1$_{62-78}$-PfCKIIβ1. Bar = 10 μm. (F) Yeast two-hybrid, BiFC and subcellular localization analyses of the putative PfCNR1 interacting proteins. A plus (+) indicates interaction and a minus (-) indicates no interaction. (G) A neighbor-joining (NJ) tree of AGAMOUS-like proteins: TAG1 (NP_001266181), AtAG (NP_001190766), PFAG (AGS44979), TAGL1 (NP_001234187) and MPF3 (AGT21646) (H) A NJ tree of CKIIβ1-like proteins. LeCKIIβ1 (XP_004241980), LeCKIIβ1L1 (Solyc01g088740.2.1), LeCKIIβ1L2 (XP_004240067) and LeCKIIβ1L3 (Solyc10g061940.1.1) The bootstrap values (> 50) for the NJ method are shown. (I) Protein-protein interactions of some combinations of PfCNR1, PfCKIIβ1, PfCYCD2;1 and PfAG2. A minus (-) indicates no interaction.

```
C1, C2 and C3 label the CArG-boxes shown in red in the figure.

PfCYCD2;1   TTGGCAACCATGGCTAAGACATGTAAGCAATATAGGCTAAGGCA--TGAGAAGGGTTGGACAAGCTAAGTCAAGGCAAGGCAAGCTAAGG   -1388
SlCYCD2;1   TTTTCAATATTAACATATTCAGTTAATTTTCTCGAGTTAAGTTTGTTGAGACTATTTTTAAAGATCA---TAGGCTCAAATACAATAAAT   -1398

PfCYCD2;1   CTTGCCATGATAATGGCTAAGTCAAATTCGTGGGCTATATGCTACGCGAGACAAATATCTAAGAAAGTGAGCACTAATGACAAGTTGTGT   -1298
SlCYCD2;1   TCAAGTACAAAAAAGAAGAAACAATGCAGGAAACATAGCAACTAAAAAATAGTGAAAACCTACAAGAAAAAGATAAAGATTCGGTTAAGT   -1308

PfCYCD2;1   G-AAGCAGAACCTGCAAGCAAGGCCGACTGTAGGCAAGCAAAAAATTTTGGTGCCAGAAT--TTAAAGGTTGAACATGCATGTGACTTGA   -1210
SlCYCD2;1   ATAATTAGGTCAAGTAATGTTTAATGATCATATATATTCATGTCATGTGTACTATAGAATAATTTGTGATTGCG-ATCCATTTAAATT--   -1221

PfCYCD2;1   ACATATTAAGCA-ATTGGTAGTACTAATATAACCGCTTGGCTTTGTCAGCATTTCGATAGGAAGAACTTGTATTTGAATTTTATAATTCG   -1122
SlCYCD2;1   ATATACTCATTATAATAATAAAAACAACAAAGCTG-TCAACTTT-TTATTATTT-AAAAATATGAAATAAAAC---AATTAAGTGTACCC   -1136

PfCYCD2;1   AAGTTAAAATGCCTTGGCAATTGG-GATTGTCAACTGTTGGCAGATTTGTGTCATATGTTTGTCATATGGCAGGCAGTAATGGCCTATTT   -1033
SlCYCD2;1   AAGCTAATAT-TTTCACTATCTACTAACCATCCGCTAT-AATACACGTCATTCACACCCTT-TTATCTAAGGTCATGTCCTCGAAAATTT   -1050

                                                   C1
PfCYCD2;1   AGAGGCACTGGCAGGCTATCTGACAGAGAGTGTGTTAGCCTAATTTGGCTAAGTTTTGTAAGGATGTTTTTGTGCTTCCTTTGTAGCACA   -943
SlCYCD2;1   GAAGTTGCGCTATGTCTATCTAATCACTTCTCTTCCATACTTTTTCGACCTACCTTT------ACCTCTGCTTATATCTACTATAAC-CA   -967

PfCYCD2;1   AGTGAAATACAAGGTTGCAAAAGTGATTCTTGTAATCCGTGTTGGCATTGTTGTTAACTTTATAGCCTATGCAAACTAAGTTTTGAACGA   -853
SlCYCD2;1   CCTCTCACACATCCTCATCTATGATATTTGAGCATCTCCTATTCACAT-GTTTAAAACATTTCATTCT-CGTTTTCTTTATCTTGTCCAC   -879

PfCYCD2;1   AAAATATAGGGTTGAAGCCATAGGTGCATGACTGCCCTTTGGAGTCGAATTATTCTGGGT-TGACCCCATATCAATACCTATATTAAAGT   -764
SlCYCD2;1   CACGATGGCCACTCCCACCTTCTTTATAATATCCTCATATCTAATCTTATTGCTCCTTATACGGCCACTCATCCACCTCAATATC----C   -793

PfCYCD2;1   CGATTAATTAAATTTGTACCATATATGAGATATTAGGGAAAC--ACTTTCTACCAAAGTTTTTTCACACTTAGAACTACTAAGACCTCTG   -676
SlCYCD2;1   TTATTTTCACGGCATGCTCTAAACATGAAATTCTTGACTAATCAACACTCAACCCTCTACATTTATGTTTTATAACTAAAAATTGTT-TG   -703

PfCYCD2;1   ATTAAGAAAGAAATAGCTTCGTTCACTGCA-TCATATCCTTGGTAGAAATAAATATTCAAATTTACTTCATC--TTAGAGAGACTTCTTG   -589
SlCYCD2;1   ATACTACAAGACTCCAATTTATCTTTAACAATTATAACTTTA-TTGTAACATAAATACAACTTAGGATCATAAGTTTCAAAAATTCCTTT   -615

PfCYCD2;1   ATACAAACTCAAACTCATTCCAACTTAGAGATGTACTTCTCGATACGAATTCAAATTCACTTCACCTTAGAGACGAATTTCTCGATACGA   -499
SlCYCD2;1   ATTT---TTCTTATTTTTTTTAA--TAAATTCAAATAAAATGAAACGAAAT-AAATAC-TTTTGTAAGATTGTCGTATTGGCTAAAAGAA   -532

                         C2
PfCYCD2;1   ATTCAGATTCTCTC-CTTCTTAAAAATGGACTTCTCGATACA-AATTCAAATATACTCCATTTTAGAGATGGGCTTCTCGATATAAATTC   -411
SlCYCD2;1   TAGCTTATGCATGCGGTAGAACGAGGAGAAACACGTGTCACGTAAAACAACTAAGAGATATTTCTAAA---GGCGTTTCGATGAAAACAT   -445

PfCYCD2;1   AAAATATGCTCCTTCTAACAGGTGGACTTCTCGATACGGATTCAATAAAAACTCCTTCGTAGAGGCGGAGTTCTCGATACGAAATCAATA   -321
SlCYCD2;1   AGT-TCGACTGTTTTTCAAATTTCAAAAAATTTTCACCG-CCAAACATTAATT--TTCAATAAATTTCATTTTTTTTTAAATTACGAATT   -359

                                                                              C3
PfCYCD2;1   AAAAATTAAAAATAAAA-ATAAGAAAAACTCCACCCTGTGTGC-TATGTGTG-GTGTGGTTTCTTGGGCCTTTTTTGGCTCTCTTTTTCC   -234
SlCYCD2;1   AACCTTCTACCCTAATACACGTCATCCGCACCACCTTGTGTGCGTGAGAGTGTGTGTGTGTTTTTGGGCCTTTTTTTGCTCTATTTTTTC   -269

PfCYCD2;1   ACTCTTTGTGTAGTTAGGATTCCACTTAGAATCCACTGTCTT-CTCTCCCCAAAATCTCTCCTTTACCCTAAACACAAAATCTTCATTTC   -145
SlCYCD2;1   -CTCCTTGTGTTGTTAGGATTCCACTTAGAATCCACTGTCTTTCTCACCCCCAAATCTCTCCTTTACCCTAAACACAAA-TCTTCATTTC   -181

PfCYCD2;1   ATTTTCCTACATAAACCTCCACTTCTTGGAATAGTTCCAATCCCCCACCAC-----------TAACCCTCCCCCTCCCTAGACACCCC--   -68
SlCYCD2;1   ATTTTCCTACATAAACTTCTTCTTCTTTGAGTAGTCTCCAGCCCTCACCTCACTTTTCTCCCTACCCCCACCCCACCCCACACCCACAAT   -91

PfCYCD2;1   -----------CACCCCAATCTTTCAAAAATGAGATTTTGAT----------AAAGGGTACATTGAGGGGTTCTTTGCTTTTGCTTTA-   -1
SlCYCD2;1   GGAAATGCTAAACACCCAAAAATTCTAAAAGAGGAGTCTTTTTGTTGTTCTTGAAAAGATACATTGAGGGGTTTTTTGATTTTGGTTTAA   -1
```

Figure S8. Promoter alignment of *SlCYCD2;1* and *PfCYCD2;1* .
These two promoters shared 48.2% identity. Around 300 bp beyond the translation initiation site marked in grey were highly conserved (71.7%) between the two promoters. The upstream regions diverged with 41.7% sequence identity. CArG-box, as the binding site for MADS-domain transcription factors, is highlighted in red. Three CArG-boxes in the *PfCYCD2;1* promoter are labeled as C1, C2 and C3. Thus, the two promoters diverged in *Solanum* and *Physalis*.

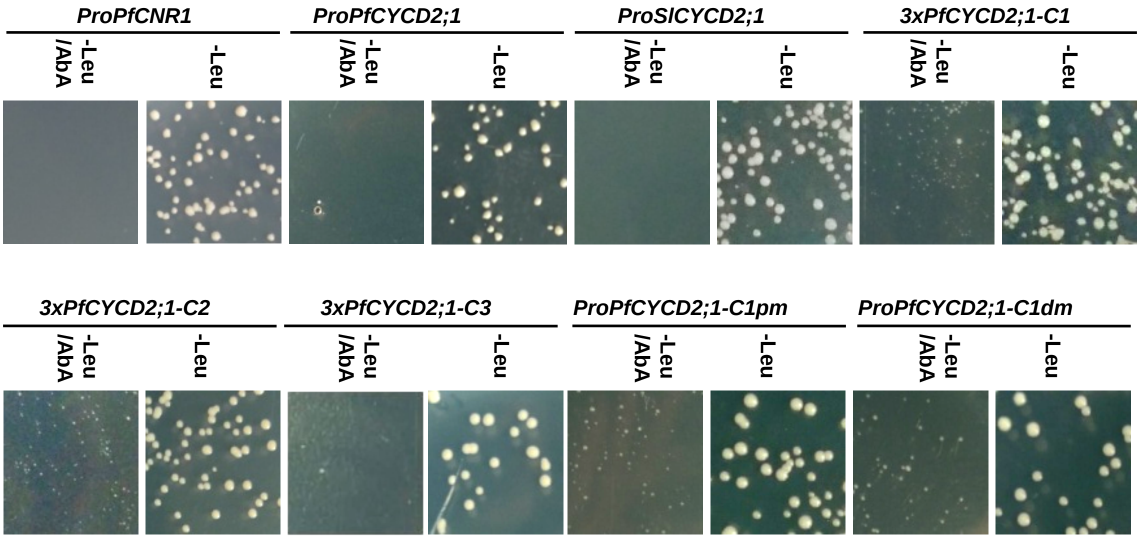

Figure S9. Yeast one-hybrid assays between PfSEP1 and the indicated DNA fragments.
Pro: Promoter; 3x indicates three tandem repeats of CArG-box. C1: CArG-box 1; C2: CArG-box 2; C3: CArG-box 3; dm: deletion mutation; pm: point mutation.

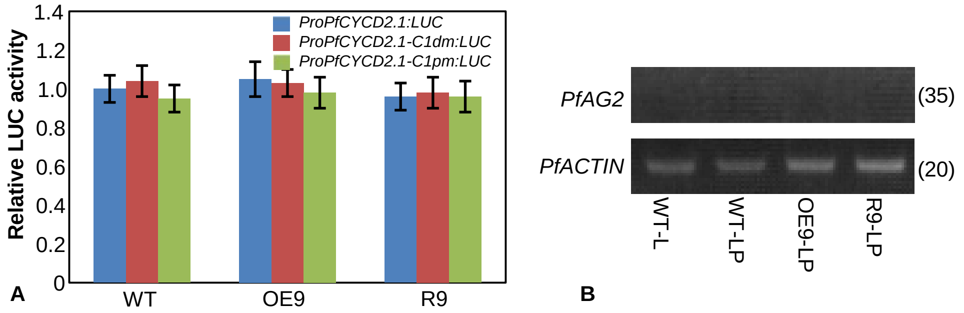

Figure S10. The LUC expression driven by the *PfCYCD2;1* promoter in *Physalis* leaf protoplasts. (A) LUC relative activity assay in *P. floridana* protoplasts of wild type (WT), *35S:PfCNR1* (OE9) and *35S:PfCNR1*-RNAi (R9). The LUC activities were normalized using the *35S:GUS* as an internal control. The mean and standard deviation from at least six independently repeated assays are presented. There is no significant differences ($P > 0.05$) compared with *ProPfCYCD2;1:LUC* in WT. (B) The expression of *PfAG2*. WT-L, the young leaves of wild type *P. floridana* (the leaf length < 1 cm); WT-LP, the protoplasts from WT-L; OE9-LP, the protoplasts from young leaves of *35S:PfCNR1* line 9; R9-LP, the protoplasts from young leaves of *35S:PfCNR1*-RNAi line 9. The total RNA from these material was subjected to RT-PCR, and a typical gel image is presented. *PfACTIN* was used as a loading control. The cycle number of PCR is given in parenthesis.

Table S1. *Physalis* resources used in the present work.

Species	Accession	Source
P. pubescens	P071	BGN
P. coztomatl	P019	BGN
P. floridana	P106	MPIZSB
P. angulata	P015	BGN
P. coztomatl	P018	BGN
P. lanceifolia	P035	BGN
P. mendocina	P037	BGN
P. pruinosa	P069	BGN
P. viscosa	P104	BGN
P. fuscomaculata	P031	BGN
P. peruviana	P044	PGRU
P. Ixocarpa	P034	BGN
P. aequata	P006	BGN
P. curassavica	P025	BGN
P. mexicana	P038	BGN
P. philadelphica	P058	PGRU
P. philadelphica	P064	PGRU

BGN, Botanical and Experimental Garden of Radbound University, Nijmegen, Netherland; PGRU, Plant Genetic Resources Unit, USDA-ARS; MPIZSB, GenBank of Max-Planck Institute for Plant Breeding Research.

Table S2. Primers used in the present work.

Usage	Name	Forward sequence (5'-3')	Reverse sequence (5'-3')
Full-length ORF	PfCNR1	ATGTATCCATCATCCACTCATC	TCACCTGATCATGCCTGCATG
	PfCNR1L1	ATGAATCCCTCAGCTCCACC	TTATCTAGTCATTCCTCCTTGAAC
	PfCNR1L2	ATGAAGTCTTCAACAAGTTC	TTATCTATACATTCCACCTTC
	PfSEP1	ATGGGAAGGGGAAGAGTAGA	TCAAACAAGTTGGAGAGGGGTT
	PfAG2	ATGGGAAGAGGAAAGATCGAG	TCAAACAAGTTGGAGAGGGGTT
	PfGIP	ATGGGGAAACTTGTTTCATT	TTAAGGGCACTTGGGTCCTC
	PfCK2B1	ATGTACAAAGAAAGAAGAG	TCAAGGCTTGTGGACCTTGA
	PfCYCD2;1	ATGGCTAAGAATAGTTATGATTC	TCAAGTCCCCTCCTCTAAAGATGTG
RT-PCR	Actin	AACTGGAATGGTCAAGGCTGG	CCATATCATCCCAATTGCTGAC
	PfCNR1	CCTAGCCTATATTCGTGCTTC	TTAGCTTGCCACCCTATTCC
	PfCNR1L1	TCTCCGATGTTCCAAACTGTTG	TTGCTCCACAAGAAGTCGTTC
	PfCNR1L2	ACAAGGGTTCCAATTCATGTG	CGATTTTTGAGCTCACGGTAC
	PfSEP1	AGTATCAACGTTGCAGCTATGC	TGAGCAAGCTGATCCAGCATG
	PfAG2	AATAGGCAAGTCACCTTCTG	ATTAGCTTCAGCGACAGATCC
	PfGIP	TATGGTCCAGGAAGCTTGAAG	TTGGTCTTCCAGTTGTTGTAG
	PfCK2B1	TCCTTCTACCTCAACTACTACTG	ACAAGACTGCTTAGACCGCACAG
	PfCYCA2-1	ACACTCTTTACCTGACTGTAC	TTCCAGTTCAACAGAGGGAACC
	PfCYCB1-1	TACCTCACAGTCAACATAATGG	TCATGTTCTCCATCTCTTGATC
	PfCYCB2-1	TGGAACAAACTGAGACAACATC	TGAGATCGAACTTGTGATGTAC
	PfCYCD2-1	ATACACCTTGCACATTCATCG	TCAAGACACTTCATCACTCTG
	PfCYCD3-1	AAGTGGAGGAGACCCAAGTTC	AGTTGCAATAACAGATGGAG
RNA *in situ* hybridization	Anti-PfCNR1-Probe	ATCACTTTCGGACAGATCTCTG	TAATACGACTCACTATAGGGTTAGCTTGCCACCCTATTCC
	Sense-PfCNR1-Probe	TAATACGACTCACTATAGGGATCACTTTCGGACAGATCTCTG	TTAGCTTGCCACCCTATTCC
Overexpression construct	pRT100-PfCNR1	CGCTCGAGAAATGTATCCATCATCCACTCATC	CGGGATCCAACTGAAGGTAGCATACTACTG
	BAR	TGAAGTCCAGCTGCCAGAAAC	GAGTCGACCGTGTACGTCTC
RNAi construct	RNAi-PfCNR1	CGACTAGTGGCGCGCCTATGGGAATAGGGTGGCAAGC	CGGGATCCATTTAAATAGTAGCACATCACCAGCTGAAG
	HYG	TACACAGCCATCGGTCCAGAC	TGGCTTGTATGGAGCAGCAGAC
VIGS assays	VIGS- PfCNR1	CGCCATGGTATGGGAATAGGGTGGCAAGC	CGCCATGGACTTCATGTGCGAGTAGAGGTG
	VIGS- PfCNR1L1	CGCCATGGATGAATCCCTCAGCTCCACCAG	CGGGATCCTTATCTAGTCATTCCTCCTTG
	VIGS- PfCNR1L2	CGCCATGGATGAAGTCTTCAACAAGTTC	CGGGATCCTTGAGCTCACGGTACTCTTGG
Yeast two-hybrid screen	Bait-PfCNR1	CGCCATGGAAATGTATCCATCATCCACTCATC	CGGGATCCTCACCTGATCATGCCTGCATG
	Bait-PfCNR1(1-42)	CGCCATGGAAATGTATCCATCATCCACTCATC	CGGGATCCCTGCGTAGTGGACGCATGCAC
	Bait-PfCNR1(103-175)	CGCCATGGAATCGTGCTTCTATAGGTCCAA	CGGGATCCTCACCTGATCATGCCTGCATG
Yeast co-transformation	Bait(Prey)-PfCNR1	CGCCATGGAAATGTATCCATCATCCACTCATC	CGGGATCCTCACCTGATCATGCCTGCATG
	Bait(Prey)-PfCNR1(1-42)	CGCCATGGAAATGTATCCATCATCCACTCATC	CGGGATCCCTGCGTAGTGGACGCATGCAC
	Bait(Prey)-PfCNR1(103-175)	CGCCATGGAATCGTGCTTCTATAGGTCCAA	CGGGATCCTCACCTGATCATGCCTGCATG
	Bait(Prey)-PfCNR1(62-78)	CGCCATGGAATGCCCTTGTATCACTTTCG	CGGGATCCTCCTTTGTTTAGTATTTCAG
	Bait(Prey)-PfGIP	CGCCATGGGGAAACTTGTTTCATT	CGGGATCCTTAAGGGCACTTGGGTCCTC
	Bait(Prey)-PfAG2	CGCCATGGGAAGAGGAAAGATCGAG	CGGGATCCTCAAACAAGTTGGAGAGGGGTT
	Bait(Prey)-PfSEP1	CGCCATGGGAAGGGGAAGAGTAGA	CGGGATCCTTAAAGCATCCACCCATGAAT
	Bait(Prey)-PfCK2β1	CGCCATGGAAATGTACAAAGAAAGAAGAG	CGGGATCCAGGCTTGTGGACCTTGA

Table S2. Primers used in the present work (continued).

Usage	Name	Forward sequence (5'-3')	Reverse sequence (5'-3')
Subcellular localization	PfCNR1-GFP	CGCAAGCTTTTATGTATCCATCATCCACTC	CGCGGATCCCCTGATCATGCCTGCATG
	PfAG2-GFP	CGCAAGCTTTTATGGGAAGAGGAAAGATCG	CGCACTAGTAACAAGTTGGAGAGGGGTTTG
	PfAG2M-GFP	CGCAAGCTTTTATGGGAAGAGGAAAGATCGAGATTGAACTC TCTGTTTTATGTGATGC	CGCACTAGTAACAAGTTGGAGAGGGGTTTG
	PfSEP1-GFP	CGCAAGCTTTTATGGGAAGGGGAAGAGTAGA	CGCACTAGTAAGCATCCACCCATGAAT
	PfCK2β1-GFP	CGCAAGCTTTTATGTACAAAGAAAGAAGAG	CGCACTAGTAGGCTTGTGGACCTTGA
	PfCNR1-N-GFP	CGCAAGCTTTTATGTATCCATCATCCACTC	CGCGGATCCCGTAGTGGACGCATGCAC
	PfCNR1-M1-GFP	CGCAAGCTTTTCAGTGGTCAACCGGTCTT	CGCGGATCCAACACAAGTAACTAAACAG
	PfCNR1-P-GFP	CGCAAGCTTTTTGCCCTTGTATCACTTTC	CGCGGATCCTCCTTTGTTTAGTATTTC
	PfCNR1--M2-GFP	CGCAAGCTTTTACAACTTCATGTGCGAG	CGCGGATCCGCACGAATATAGGCTAGG
	PfCNR1--C-GFP	CGCAAGCTTTTTTCTATAGGTCCAAAATG	CGCGGATCCCCTGATCATGCCTGCATG
	PfCNR1-NM1P-GFP	CGCAAGCTTTTATGTATCCATCATCCACTC	CGCGGATCCTCCTTTGTTTAGTATTTC
	PfCNR1-PM2C-GFP	CGCAAGCTTTTTGCCCTTGTATCACTTTC	CGCGGATCCCCTGATCATGCCTGCATG
	Mutation-NP	GTCCACTACGTGCCCTTGTATCACTTTC	TACAAGGGCACGTAGTGGACGCATGCAC
	Mutation-PC	AAACAAAGGATTCTATAGGTCCAAAATG	ACCTATAGAATCCTTTGTTTAGTATTTC
BiFC	PfCNR1-YFPn(YFPc)	CGACTAGTATGTATCCATCATCCACTCA	CGGGATCCCCTGATCATGCCTGCATGAC
	YFPn(YFPc)-PfCNR1	CGACTAGTATGTATCCATCATCCACTCA	CGGGATCCCCTGATCATGCCTGCATGAC
	PfCNR1-N-YFPn	CGACTAGTATGTATCCATCATCCACTCA	CGCGGATCCCTGCGTAGTGGACGCATGCAC
	YFPn-PfCNR1-N	CGACTAGTATGTATCCATCATCCACTCA	CGCGGATCCCTGCGTAGTGGACGCATGCAC
	PfCNR1-M1-YFPn	CGACTAGTCAGTGGTCAACCGGTCTT	CGCGGATCCAACACAAGTAACTAAACAG
	YFPn-PfCNR1-M1	CGACTAGTCAGTGGTCAACCGGTCTT	CGCGGATCCAACACAAGTAACTAAACAG
	PfCNR1-M2-YFPn	CGACTAGTACAACTTCATGTGCGAG	CGCGGATCCGCACGAATATAGGCTAGG
	YFPn-PfCNR1-M2	CGACTAGTACAACTTCATGTGCGAG	CGCGGATCCGCACGAATATAGGCTAGG
	PfCNR1-C-YFPn	CGACTAGTTCGTGCTTCTATAGGTCC	CGGGATCCCCTGATCATGCCTGCATGAC
	YFPn-PfCNR1-C	CGACTAGTTCGTGCTTCTATAGGTCC	CGGGATCCCCTGATCATGCCTGCATGAC
	PfCNR1-P-YFPn	CGCACTAGTTGCCCTTGTATCACTTTCG	CGCGGATCCTCCTTTGTTTAGTATTTCAG
	YFPn-PfCNR1-P	CGCACTAGTTGCCCTTGTATCACTTTCG	CGCGGATCCTCCTTTGTTTAGTATTTCAG
	PfCNR1-NM1P-YFPn	CGCGGATCCATGTATCCATCATCCACTC	CGCCTCGAGTCCTTTGTTTAGTATTTC
	YFPn-PfCNR1-NM1P	CGCGGATCCATGTATCCATCATCCACTC	CGCCTCGAGTCCTTTGTTTAGTATTTC
	PfCNR1-PM2C-YFPn	CGCGGATCCTGCCCTTGTATCACTTTC	CGCCTCGAGCCTGATCATGCCTGCATG
	YFPn-PfCNR1-PM2C	CGCGGATCCTGCCCTTGTATCACTTTC	CGCCTCGAGCCTGATCATGCCTGCATG
	PfCNR1-NPC-YFPn	CGACTAGTATGTATCCATCATCCACTCA	CGGGATCCCCTGATCATGCCTGCATGAC
	YFPn-PfCNR1-NPC	CGACTAGTATGTATCCATCATCCACTCA	CGGGATCCCCTGATCATGCCTGCATGAC
	PfGIP-YFPn(YFPc)	CGACTAGTATGGGGAAACTTGTTTCA	CGGGATCCAGGGCACTTGGGTCCTCCTTC
	PfSEP1-YFPn(YFPc)	CGACTAGTATGGGAAGGGGAAGAGTAGA	CGGGATCCAAGCATCCACCCATGAAT
	PfAG2-YFPn(YFPc)	CGACTAGTATGGGAAGAGGAAAGATCGAG	CGGGATCCAACAAGTTGGAGAGGGGTTTG
	PfCK2β1-YFPn(YFPc)	CGACTAGTATGTACAAAGAAAGAAGAG	CGGGATCCAGGCTTGTGGACCTTGA
Yeast One-Hybrid	Promotor-PfCYCD2	CGCAAGCTTAATGCCTTGGCAATTGGGATTG	CGCCTCGAGAAAGGTCTGAGACTACAGAATC
	Promotor-CArG1	AGCTTCCTAATTTGGCCTAATTTGGCCTAATTTGG	TCGAGCCAAATTAGGCCAAATTAGGCCAAATTAGG
	Promotor-CArG2	AGCTTCTTAAAAATGCTTAAAAATGCTTAAAAATG	TCGAGCATTTTTAAGCATTTTTAAGCATTTTTAAG
	Promotor-CArG3	AGCTTCCTTTTTTGGCCTTTTTTGGCCTTTTTTGG	TCGAGCCAAAAAAGGCCAAAAAAGGCCAAAAAAGG
	Promotor-SlCYCD2	CGCAAGCTTACAATGCAGGAAACATAGCAAC	CGCCTCGAGTACCCAAAGAACCAAGATCATC
	Promotor-PfCNR1	CGCAAGCTTAACCTTTGAATACACCACCAGA	CGCCTCGAGAGTCTTGATTAGTGGATGATGG
	Promotor-CYCD-dm	AGTGTGTTAGCTAAGTTTTGTAAGGATG	CAAAACTTAGCTAACACACTCTCTGTCA
	Promotor-CYCD-pm	AGTGTGTTAGAATAATTTAACTAAGTTTTGTAAGGATG	CAAAACTTAGTTAAATTATTCTAACACACTCTCTGTCA
LUC activity assay	yy96-CYCD2;1	CGCAAGCTTAATGCCTTGGCAATTGGGATTG	CGCGGATCCAAAGGTCTGAGACTACAGAATC
	CYCD-dm	AGTGTGTTAGCTAAGTTTTGTAAGGATG	CAAAACTTAGCTAACACACTCTCTGTCA
	CYCD-pm	AGTGTGTTAGAATAATTTAACTAAGTTTTGTAAGGATG	CAAAACTTAGTTAAATTATTCTAACACACTCTCTGTCA
	221-AG2	CGCTCTAGAATGGGAAGAGGAAAGATCGAG	CGCGGTACCAACAAGTTGGAGAGGGGTTTG
	221-AG2M	CGCTCTAGATCTTCTTCCCAGAAGAAAATG	CGCGGTACCAACAAGTTGGAGAGGGGTTTG
	221-SEP1	CGCTCTAGAATGGGAAGGGGAAGAGTAGAAC	CGCGGTACCAAGCATCCACCCATGAATAAATC
	221-PfCNR1	CGCTCTAGAATGTATCCATCATCCACTCATC	CGCGGTACCCCTGATCATGCCTGCATGACA
	221-CK2	CGCTCTAGAATGTACAAAGAAAGAAGAGGTG	CGCGGTACCAGGCTTGTGGACCTTGAAACC

Table S3. Variation in cell number in the ovaries and berries during development.

		Cell number						Cell division activity (cell cycle number)						
		B1	B2	B3	F	5DPA	50DPA	B1-B2	B2-B3	B3-F	B1 (B2)-F	F-5DPA	5DPA-50DPA	F-50DPA
Pericarp	P058	512±71	1205±150	1558±172	1567±170	1642±195		1.23	0.37	0.01	3.06	0.07		
	P064	507±69	1099±137	1501±164	1512±132	1550±231		1.12	0.45	0.01	2.98	0.04		
	P106	534±79	554±55	593±87	943±113	1078±136		0.05	0.1	0.67	1.77	0.19		
Placenta	P058	406±64	1472±189	2066±215	2163±155	2268±287		1.86	0.49	0.07	5.33	0.07		
	P064	383±64	1290±128	1910±184	1925±140	2055±217		1.75	0.57	0.01	5.03	0.09		
	P106	386±58	511±56	642±98	1107±124	1317±172		0.4	0.33	0.79	2.87	0.25		
Flesh	P058	915 ±95	2670 ±290	3624 ±340	3730 ±270	3910±415	4180 ±420	1.54	0.44	0.04	2.03	0.07	0.1	0.16
	P064	890 ±110	2390 ±220	3410 ±285	3437 ±260	3605±360	4140 ±360	1.43	0.51	0.01	1.95	0.07	0.2	0.27
	P106	920 ±85	1065 ±95	1235 ±150	2050 ±170	2395±250	2860 ±280	0.21	0.21	0.73	1.16	0.22	0.26	0.48
Ovule	P058		43 ±8	176 ±20	270 ±35	290 ±30	7780 ±880		2.03	0.62	2.65	0.1	4.75	4.85
	P064		40 ±6	137 ±11	263 ±25	275 ±25	6065 ±550		1.78	0.94	2.72	0.06	4.46	4.53
	P106		35 ±5	115 ±11	200 ±20	205 ±18	3023 ±360		1.72	0.8	2.51	0.04	3.88	3.92

The cell number in ovaries from the flower buds at B1, B2 and B3 stages; mature flowers (F) as well as 5- and 50-DPA (day post-anthesis) berries (see Methods) were investigated in *P. philadelphica* (P058), *P. philadelphica* (P064) and *P. floridana* (P106). Flesh = Pericarp + Placenta. Average cell cycle (C), for example, from B1 to B2 was defined as $\log_2$ [number (B2) / number (B1)]. The C values between other developmental stages were similarly estimated.

Table S4. Variation of cell size in the ovaries and the berries during development.

		Cell size (um^2)						Cell expansion degree						
		B1	B2	B3	F	5DPA	50DPA	B1-B2	B2-B3	B3-F	B1 (B2)-F	F-5DPA	5DPA-50DPA	F-50DPA
	P058	92±9	72±10	178±16	288±35	403±33		0.78	2.47	1.62	3.13	1.4		
Pericarp	P064	86±8	76±12	186±18	218±28	385±35		0.88	2.45	1.17	2.53	1.77		
	P106	91±10	81±10	110±12	120±12	265±25		0.89	1.36	1.1	1.32	2.21		
	P058	89±9	55±8	135±15	213±31	275±20		0.62	2.45	1.58	2.39	1.29		
Placenta	P064	89±7	58±11	142±15	162±25	312±24		0.65	2.45	1.14	1.82	1.93		
	P106	87±8	75±12	90±11	93±15	204±18		0.86	1.2	1.03	1.07	2.19		
	P058	91 ±10	60 ±8	150±13	245 ±25	340 ±32	79590±8530	0.66	2.5	1.63	2.69	1.39	234.09	324.86
Flesh	P064	88 ±8	65 ±10	160±13	183 ±20	315 ±29	29690±3200	0.74	2.46	1.14	2.08	1.72	94.25	162.24
	P106	90 ±10	78 ±12	95 ±12	105 ±15	230 ±25	9540 ±980	0.87	1.22	1.11	1.17	2.19	41.48	90.86
	P058		40 ±6	48 ±6	55 ±7	70 ±9	410 ±55		1.20	1.15	1.38	1.27	5.85	7.45
Ovule	P064		30 ±5	35 ±5	42 ±5	65 ±6	309±40		1.17	1.20	1.40	1.55	4.75	7.36
	P106		22 ±4	30 ±4	35 ±4	60 ±7	459 ±45		1.36	1.17	1.60	1.71	7.65	13.11

The cell size in the ovaries from the flower buds at B1, B2 and B3 stages; mature flowers (F) as well as 5- and 50-DPA (day post-anthesis) berries (see Methods) were investigated in *P. philadelphica* (P058), *P. philadelphica* (P064) and *P. floridana* (P106). Flesh = Pericarp + Placenta. Average cell expansion degree from B1 to B2 was defined as size (B2) / size (B1); the expansion degrees between other developmental stages were similarly estimated.

Table S5. Correlation between *PfCNR1*-like expression and ovary cell activities.

	Cell cycle number	Cell expansion degree	Relative mRNA accumulation (RMA)		
			PfCNR1	***PfCNR1L1***	***PfCNR1L2***
P058	2.03	2.69	1.34±0.09	4.44±0.23	6.40±0.18
P064	1.95	2.08	1.63±0.12	25.29±2.28	7.17±0.72
P106	1.16	1.17	12.74±0.28	29.95±2.29	5.14±0.57
Correlation (cell cycle and RMA)			*r* = -0.998*<br>*P* = 0.039	*r* = -0.703<br>*P* = 0.504	*r* = 0.892<br>*P* = 0.298
Correlation (cell expansion and RMA)			*r* = -0.926<br>*P* = 0.247	*r* = -0.894<br>*P* = 0.296	*r* = 0.700<br>*P* = 0.506

P. philadelphica (P058), *P. philadelphica* (P064) and *P. floridana* (P106) were investigated. Average cell cycle and cell expansion degree in the ovaries (B1-F) are available in Table S3 and S4; Relative mRNA accumulation (RMA) of each *PfCNR1*-like gene before anthesis was defined as [expression (B1) + expression (B2) + expression (B3) + expression (F)] (Figure 3C; Figure S3). Significance was defined as $P < 0.05$ and highlighted with a star (*).

Table S6. Variation in sequences and expressions, and their correlations with organ size within *Physalis.*

	Amino acid variation of PfCNR1									Gene expression	
	7	29	34	42	84	93	148	160	173	*PfCNR1*	*PfCYCD2;1*
P. pubescens-P071	N	P	M	Q	S	G	M	S	T	9.80±0.75	2.00±0.12
P. coztomatl-P019	N	S	M	Q	S	G	I	S	T	11.09±0.74	5.71±0.46
P. floridana-P106	H	P	M	Q	S	G	M	S	I	10.64±0.81	1.00±0.09
P. angulata-P015	N	P	M	Q	S	G	M	N	T	9.18±0.78	7.14±0.74
P. coztomatl-P018	N	P	M	Q	S	G	M	S	T	8.99±0.80	2.86±0.24
P. lanceifolia-P035	N	P	V	Q	S	G	M	S	T	9.15±0.75	8.14±0.94
P. mendocina-P037	N	P	M	P	S	G	M	N	T	3.01±0.26	5.24±0.43
P. pruinosa-P069	N	P	V	P	S	A	M	S	T	2.91±0.19	6.35±0.70
P. viscosa-P104	H	P	M	Q	N	G	I	N	I	2.65±0.16	1.27±0.15
P. fuscomaculata-P031	N	T	M	Q	S	G	M	S	T	2.63±0.18	7.94±0.80
P. peruviana-P044	N	P	M	Q	S	G	M	S	T	2.30±0.14	5.29±0.51
P. ixocarpa-P034	N	P	V	Q	S	A	M	S	I	2.00±0.23	7.94±0.74
P. aequata-P006	N	P	M	P	S	A	I	S	T	1.99±0.21	6.35±0.66
P. curassavica-P025	H	P	V	Q	S	A	M	S	T	1.06±0.12	9.29±1.01
P. mexicana-P038	N	P	M	Q	S	G	M	S	T	1.00±0.11	7.30±0.89
P. philadelphica-P058	N	P	M	Q	S	G	M	S	T	1.52±0.14	10.48±1.11
P. philadelphica-P064	N	P	V	Q	S	G	M	S	T	1.89±0.14	9.52±0.85
P values (flower length)	0.64	0.36	0.11	0.76	0.33	0.92	0.62	0.41	0.48	Correlation of *PfCNR1-organ size*<br>*r* = -0.45, *P* = 0.07 (flower length)<br>*r* = -0.67**, *P* = 0.00 (berry weight)<br>*r* = -0.78**, *P* = 0.00 (seed weight)	
P values (fruit weight)	0.79	0.62	0.35	0.81	0.61	0.16	0.82	0.24	0.62	Correlation of *PfCYCD2;1*-organ size<br>*r* = -0.10, *P* = 0.69 (flower length)<br>*r* = 0.65**, *P* = 0.00 (berry weight)<br>*r* = 0.49*, *P* = 0.04 (seed weight)	
P values (seed weight)	0.68	0.59	0.79	0.94	0.63	0.11	0.77	0.55	0.75	Correlation of *PfCNR1-PfCYCD2;1*<br>*r* = -0.50* *P* = 0.04	

The *P* value for the correlation between amino acid and organ size is > 0.05, indicating that amino acid variation did not account for organ size variation. In the correlation of gene expression and organ size, a star (*) indicates significance at *P* < 0.05; two stars (**) indicate significance at *P* < 0.01.

Table S7. Phenotypic variation of *35S:PfCNR1*-RNAi and *35S:PfCNR1* transgenic *Physalis* plants.

		Leaf size (mm^2)	Flower length (mm)	Carpel weight (mg)	Sepal size (mm^2)	Fruit weight (g)	ICS size (mm^2)	100-seed weight (mg)	Plant weight (g)	Fruit yield per plant (g)	Seed number per fruit
**WT**	**WT**	2320±210	8±0.7	3±0.4	20±1.9	0.8±0.1	1375±105	40±1	22.1±2.6	23.2±4.6	120±15
**RNAi**	R1	2470±170	9.2±0.5*	4.6±0.35**	23±1.5*	0.98±0.08*	1480±120	40±0.9	21.3±2.8	24±4.8	135±18
	R2	2450±150	9.5±0.6**	4.5±0.36**	23.5±2*	1.02±0.09*	1450±100	42.2±1.2*	20.5±3.8	26.6±3.8	140±20
	R3	2520±180	9.8±0.5**	4.8±0.35**	25±2**	1.06±0.1**	1500±150	42.4±1.1**	22.6±2.3	18±3.2	110±17
	R4	2750±210*	10.5±0.7**	5±0.4**	26.5±2.2**	1.18±0.11**	1530±145	43.6±1.3**	24±3.2	21.6±3.8	120±18
	R5	2880±200**	11.2±0.6**	5.2±0.36**	26±2.4**	1.1±0.13**	1565±125*	43.1±1.1**	23.5±3.3	26.4±4.2	110±12
	R6	2960±230**	11±0.5**	5.5±0.38**	27±1.9**	1.22±0.12**	1580±130*	45.3±1.5**	23±2.1	24.8±3.2	105±13
	R7	3250±220**	12.1±0.8**	5.4±0.48**	27±3.2**	1.2±0.13**	1560±155*	47.3±1.7**	24.5±3.5	19.6±2.4	115±17
	R8	3360±240**	12±0.7**	5.6±0.4**	28±2.2**	1.2±0.1**	1600±175*	46.1±2**	22±2.2	27±4.5	120±18
	R9	3680±280**	12.8±0.8**	5.8±0.44**	28±2.4**	1.26±0.14**	1620±160**	47.3±1.9**	19.5±3.6	22±3.4	135±22
	PC	*r* = -0.805<br>*P* = 0.005	-0.917<br>1.8×10^{-4}	-0.987<br>1.1×10^{-7}	-0.961<br>9.6×10^{-6}	-0.944<br>4×10^{-5}	-0.931<br>8.6×10^{-5}	-0.829<br>0.003	-0.104<br>0.776	-0.001<br>0.998	0.123<br>0.736
**OE**	OE1	2180±100	7.3±0.2**	2.95±0.19	18.5±1*	0.73±0.06	1305±75	38.3±0.8*	22.2±3.1	29±4.2	135±20
	OE2	2080±95*	7.3±0.3**	2.84±0.18*	18.3±0.8*	0.7±0.06*	1320±70	38.2±0.9*	20.5±3.8	26.6±3.8	135±18
	OE3	2200±100	7±0.3**	2.86±0.2*	19.1±0.8	0.71±0.06*	1255±70*	38.8±0.9	24.3±3.5	18±3.2	110±13
	OE4	1750±90**	7.4±0.2**	2.63±0.13**	18±0.9**	0.67±0.05**	1235±65**	37.6±0.8**	24±3.2	19±3	115±10
	OE5	1830±85**	6.7±0.3**	2.71±0.2**	17.6±0.7**	0.7±0.07*	1215±80**	37.2±0.7**	19.5±2.6	27±5.3	115±13
	OE6	1810±90**	6.9±0.4**	2.64±0.18**	17.9±0.8**	0.68±0.05**	1210±65**	36.8±0.8**	22±2.3	24.8±5.1	110±10
	OE7	1500±70**	6.6±0.2**	2.42±0.16**	17.5±1**	0.67±0.06**	1250±70*	36.5±0.8**	24±3.5	19±3.8	115±13
	OE8	1535±80**	7±0.5**	2.45±0.18**	18±0.7**	0.68±0.07**	1190±65**	37.3±0.9**	23±4.2	24±3.8	120±22
	OE9	1510±75**	6.8±0.4**	2.56±0.17**	17.6±0.8**	0.68±0.05**	1210±65**	36.4±0.7**	19.1±2.8	23.8±4.1	135±23

Pearson correlation (PC) between *PfCNR1* expression and organ size in 9 lines of the *35S:PfCNR1*-RNAi was evaluated. The correlation coefficients (*r*) and the *P* values are presented. The differential significance of the organ size in transgenic plants compared to wild type organs was evaluated using a two-tailed *t*-test. A star (*) indicates significance at $P < 0.05$; two stars (**) indicate significance at $P < 0.01$.

Table S8. Cells in *35S:PfCNR1*-RNAi and *35S:PfCNR1* transgenic *Physalis* plants.

	**Leaf**			**Sepal**			**ICS**			**Pericarp**		
	**WT**	**OE9**	**R9**	**WT**	**OE9**	**R9**	**WT**	**OE9**	**R9**	**WT**	**OE9**	**R9**
Cell number	849688 ±35836	549121 ±40472	1327110 ±39257	26882± 570	23770 ±356	35788± 2076	658852 ±14784	569501 ±14899	781735 ±15429	990 ±60	812 ±46	1455 ±80
P (*t*-test)		0.000 **	0.000 **		0.000 **	0.000 **		0.000 **	0.000 **		0.000 **	0.000 **
Cell size (μm^2)	2680 ±76	2722 ±57	2736 ±93	740 ±67	788 ±62	750 ±46	2144 ±133	2088 ±124	2130 ±126	119 ±7	122 ±7	117 ±8
P (*t*-test)		0.354	0.328		0.272	0.790		0.511	0.869		0.461	0.711

	**Placenta**			**Ovule**			**Flesh**			**Seed**		
	**WT**	**OE9**	**R9**	**WT**	**OE9**	**R9**	**WT**	**OE9**	**R9**	**WT**	**OE9**	**R9**
Cell number	1072 ±58	916 ±45	1929 ±89	201 ±12	155 ±8	278 ±14	2853 ±79	2535 ±51	4667 ±173	3037 ±70	2811 ±75	3392 ±88
P (*t*-test)		0.000 **	0.000 **		0.000 **	0.000 **		0.000 **	0.000 **		0.001 **	0.000 **
Cell size (μm^2)	90 ±8	91 ±8	93 ±10	30.8 ±2.6	33 ±4.8	34.6 ±4.6	12258 ±735	11548 ±1034	10058 ±681	476 ±15	454 ±34	482 ±42
P (*t*-test)		0.842	0.585		0.397	0.144		0.263	0.001 **		0.227	0.772

Epidermal cells of leaves, sepals and ICSs were observed with scanning electron microscopy (SEM). Total cell number in these organs was estimated based on cell number per unit organ area. Cells of ovaries, ovules, flesh and seeds were measured based on the median transverse sections of mature flowers (F), mature berries (M) and mature seeds. Total cell number in a median transverse section was counted. For each section or SEM image, the sizes of 150 randomly selected cells were measured. 5 median transverse sections for each tissue or organ were investigated. The differential significance (*P* values) of cell number/size in transgenic plants compared to the wild-type organs was evaluated using a two-tailed *t*-test. Two stars (**) indicate significant difference at $P < 0.01$.
